# Development, feasibility testing and evaluation of a family-oriented mobile application to promote healthy lifestyle in infants and parents during early life: a mixed methods study

**DOI:** 10.3389/fdgth.2026.1869210

**Published:** 2026-06-19

**Authors:** Kai Ting Mok, Daniel Chan, Chengsi Ong, Ming Hui Chiew, Mei Chien Chua, Annabel Ngien, Navin Michael, Varsha Gupta, Jerry Kok Yen Chan, Fabian Yap, Chee Wai Ku, See Ling Loy

**Affiliations:** 1 Department of Reproductive Medicine, KK Women’s and Children’s Hospital, Singapore, Singapore; 2 Endocrinology Service, Department of Paediatrics, KK Women’s and Children’s Hospital, Singapore, Singapore; 3 Academic Clinical Program, Duke-NUS Medical School, Singapore, Singapore; 4 Department of Nutrition and Dietetics, KK Women’s and Children’s Hospital, Singapore, Singapore; 5 SwagSoft Studios Pte Ltd, Singapore, Singapore; 6 SingHealth Duke-NUS Maternal and Child Health Research Institute (MCHRI), KK Women’s and Children’s Hospital, Singapore, Singapore; 7 Department of Neonatology, KK Women’s and Children’s Hospital, Singapore, Singapore; 8 Institute for Human Development and Potential (IHDP), Agency for Science Technology and Research, Singapore, Singapore; 9 Human Potential Translational Research Program, Yong Loo Lin School of Medicine, National University of Singapore, Singapore, Singapore; 10 Bioinformatics Institute (BII), Agency for Science Technology and Research, Singapore, Singapore; 11 Lee Kong Chian School of Medicine, Nanyang Technological University, Singapore, Singapore

**Keywords:** digital health, family-centered care, maternal and child health, mHealth, user experience

## Abstract

**Introduction:**

Early-life health behaviors are shaped within the family environment, influencing long-term metabolic and developmental outcomes. Mobile health (mHealth) applications are widely available but most lack clinical integration, behavioral theory grounding, and personalized interactivity. This study describes the development and feasibility testing of the *FamNucleus* minimum viable product (MVP) and evaluates its usability and perceived quality among parents and healthcare professionals (HCPs) in supporting infant and parental health from a life-course behavioral perspective.

**Methods:**

*FamNucleus* was co-developed by a multidisciplinary clinical team, guided by the UK Medical Research Council framework for complex interventions, informed by the Capability, Opportunity, Motivation-Behavior model and Behavior Change Wheel. In this mixed-methods study, 25 parents and 6 HCPs used the MVP for one month (December 2024-January 2025). User experience was assessed using the User Experience Questionnaire-Key Performance Indicator (UEQ-KPI), UEQ, and user version of the Mobile App Rating Scale (uMARS). HCPs additionally completed the mHealth App Usability Questionnaire (MAUQ). Content validity was evaluated using the Content Validity Index (I-CVI). Focus group discussions were thematically analyzed using NVivo to explore user perceptions and conditions for adoption.

**Results:**

UEQ-KPI perspicuity decreased significantly among parents and HCPs from prototype to MVP. On UEQ, parents rated attractiveness, perspicuity, and stimulation above average, while efficiency, dependability, and novelty were lower. HCPs reported higher UEQ scores overall, with excellent ratings for attractiveness, perspicuity, and novelty. On uMARS, overall app quality was rated positive by both groups. Clinical usability was rated positively by HCPs on MAUQ. Content validity was acceptable (parents: ≥0.92 across domains except ambiguity at 0.80; HCPs: ≥0.83). Qualitative findings indicated that *FamNucleus* was perceived as a family-oriented caregiving platform, with generally positive initial experiences, intuitive onboarding, and appealing app interface. Participants engaged with key features, including artificial intelligence-driven Chatbot, Diary, Trackers/Survey, Enrich modules, and Nudges. Mixed usability findings and challenges related to app clarity, navigation, and sustained engagement were reported.

**Conclusion:**

*FamNucleus* demonstrated preliminary feasibility and acceptable usability as a family-centered mHealth app with perceived value in supporting early caregiving and complementing routine clinical care. Nevertheless, mixed usability findings indicate the need for further refinement prior to broader implementation.

## 1 Introduction

Early-life exposures shape long-term health trajectories. The Developmental Origins of Health and Disease (DOHaD) framework highlights how infant feeding, parental diet, sleep, stress, and caregiving environments influence metabolic and developmental outcomes across the life-course (1–3). Evidence from local longitudinal birth cohorts, such as the Growing Up in Singapore Towards healthy Outcomes (GUSTO) study further demonstrates associations between maternal nutrition, early feeding practices, and subsequent child adiposity and metabolic outcomes (4). Poor infant feeding practices and suboptimal parental nutrition can adversely affect children's health and development, contributing to micronutrient deficiencies and long-term health complications (5). Hence, improving early caregiving cannot solely focus on isolated individual behaviors, but requires approaches focused on the family-centered behavioral ecosystem.

Integration of digital health, particularly with advancements in Artificial Intelligence (AI) and Large Language Models, potentially adds values to early life interventions. The proliferation of social media and online parenting forums has created a multi-channel digital ecosystem, normalizing the sharing and documentation of children's growth, behaviors, and daily experiences (6). This presents an opportunity for meaningful engagement through structured digital tools. While maternity-related mobile apps have gained attention in recent years, evidence suggests that only a handful have interactive or personalized features (7–9). Current mobile health (mHealth) landscape remains fragmented, as the majority are commercially developed, operate independently, and unregulated by clinical governance (10). Existing apps commonly provide simplistic trackers or static information delivery, with limited integration of behavioral design theory, clinical oversight, emotional support, and social support systems (11).

Health behavior change is more sustainable when anchored in established theoretical framework, as knowledge by itself may fail to influence long-lasting behavioral change (12). The Capability-Opportunity-Motivation-Behavior (COM-B) model (13) and the Behavior Change Wheel (BCW) (14) offer systematic strategies to improve knowledge, empower self-regulation, support motivation, and redesign environmental cues. Bringing these behavioral theories to the development of app features can underpin digital health interventions to not only deliver information but also implement the structured and mechanism-based lifestyle changes. Nevertheless, few family-friendly digital health applications are directly mapping the functions of apps to proven behavioral mechanisms. Early childhood preventive health is a national priority in Singapore (15), yet no existing hospital-affiliated, integrated digital platform that can support infant and family health, while fitting in with local clinical guidelines and workflows.

To address these gaps, we developed *FamNucleus*, a clinician-led, family-oriented mHealth app to facilitate the adoption of healthy behaviors beginning with early caregiving and progressively extending to comprehensive family health and wellbeing throughout the life-course. *FamNucleus* includes functionalities that help parents make better-informed choices about infant feeding, coordinating parental care, monitor parent lifestyle and health behaviors, provide access to curated health information, and receive support through professional oversight. This paper outlines the development of the *FamNucleus* and describes the results of the feasibility evaluation, which included an analysis of the initial user experience and viability of the implementation into a real-world context. Specifically, the acceptability of the minimum viable product (MVP) was evaluated using a mixed-methods study design.

## 2 Methodology

### 2.1 Study design

**Figure 1:**
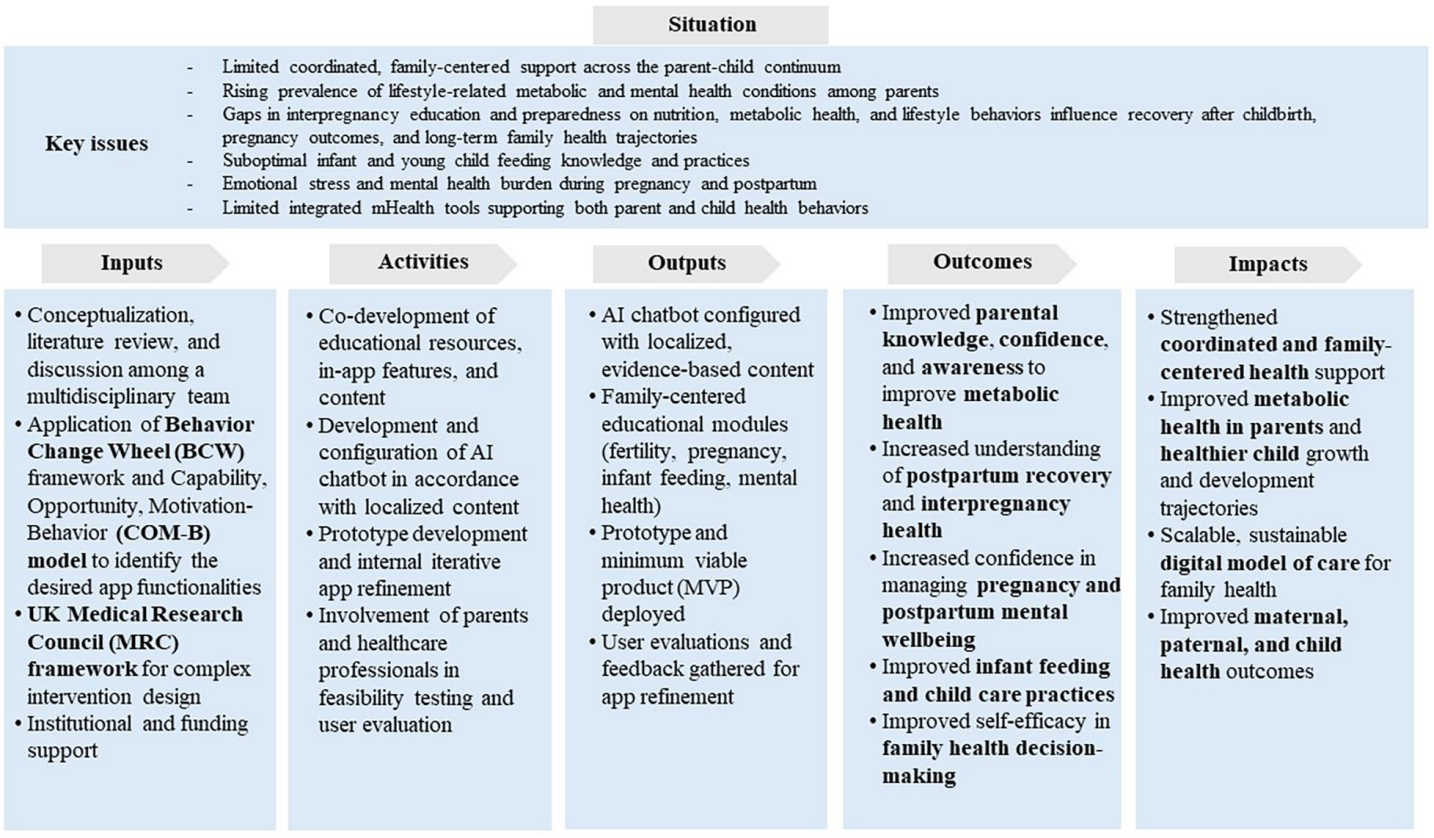
The logic model outlines the key issues as existing gaps, inputs and activities undertaken during development, intervention outputs, expected outcomes, and long-term impacts on parental metabolic health, mental wellbeing, infant feeding practices, and family health trajectories.

This paper describes the process of developing and feasibility testing of *FamNucleus*, a clinician-led, co-designed mHealth solution, which aims to support parents during the early child-rearing process. Findings from the current study will inform additional refinement of the app before its evaluation as an intervention tool in a subsequent study. The UK Medical Research Council (MRC) framework for developing and evaluating complex interventions (16) was used to structure and guide the current study design, which emphasizes a systematic, phased approach consisting of development, feasibility testing, evaluation, and implementation, with iterative refinements being made by evidence, theory, and input from stakeholders (17). A logic model was then integrated through intervention mapping to systematically translate evidence and theory into practice, mapping key inputs and activities to expected outputs and outcomes (Figure 1). The app development process was conducted from October 2023 to October 2024, and the feasibility testing was conducted between December 2024 and January 2025.

### 2.2 Development of the *FamNucleus*

#### 2.2.1 Co-design and multidisciplinary approach

*FamNucleus* was co-developed by a multidisciplinary team from KK Women's and Children's Hospital (KKH), Singapore. During the development, paediatricians, obstetricians, dietitians, and allied health professionals collaborated with the national Maternal and Child Health (MCH) stakeholders and a mobile app developer (Swagsoft Studios Pte Ltd). The core aim of this app is to address the specific needs of parents during the early infant care and parenting journey. Contributions from a multidisciplinary team helped ensure that the components of *FamNucleus* were clinically accurate, culturally relevant, and user-friendly, factoring in the app design principles.

#### 2.2.2 Needs assessment and evidence-informed design

A literature review on postpartum care, digital health interventions, and actionable behavior change was conducted prior to the app development phase to determine critical gaps and inform app design decisions. Leveraging the lived experiences of parents and HCPs, the team collaborated to conceptualize and develop the family-centered care pathways in the *FamNucleus*. Early engagements with specialists in obstetrics, pediatrics, nutrition, and digital health shaped the first iteration of the design and the scope of *FamNucleus*.

#### 2.2.3 Incorporation of behavioral theory into the app

**Figure 2:**
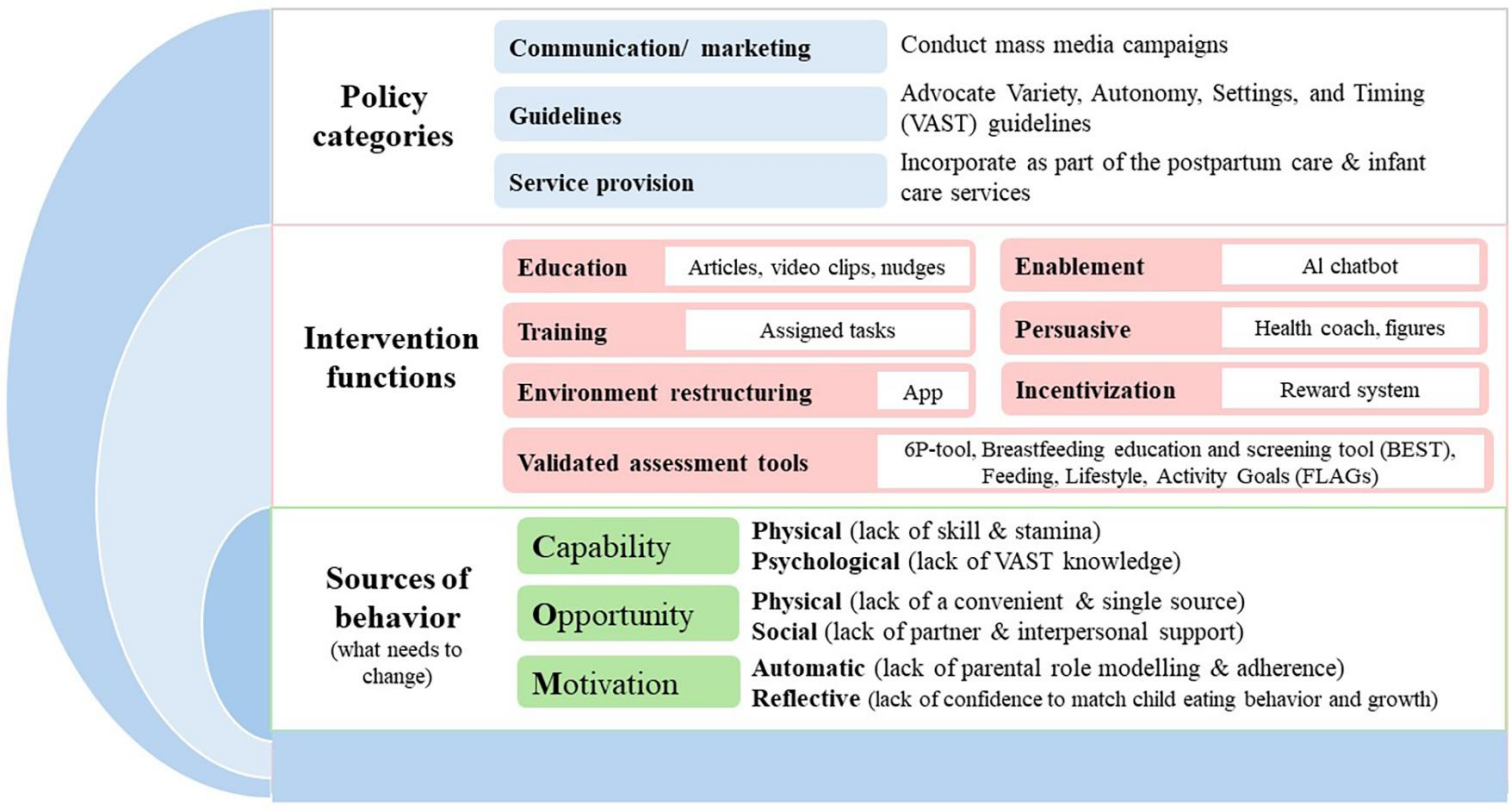
Mapping of COM-B determinants to intervention functions, policy categories, and intervention functions in the development of the FamNucleus.

The COM-B model (13) and the BCW framework (14) was used to inform the design and content of the *FamNucleus* app. These frameworks (Supplementary material A and Figure 2) guided as the basis for several intervention functions built into the app, including education, training, environment restructuring, enablement, persuasion, and incentivization. While the behavioral theories guided the design of the intervention content and intended behavioral targets, the evaluation tools used in this study were chosen to assess early-stage implementation outcomes represent essential proximal indicators before evaluating downstream behavioral outcomes in real-world settings. The current evaluation outcomes included usability, acceptability, engagement, and perceived app quality. To ensure future scalability and sustainability, additional policy options were also factored into areas, including communication or marketing, guidelines, and service provision. These combined theories may facilitate sustained engagement with the app and support measurable behavioral outcomes during the development and piloting stages. Comprehensive evaluation and implementation are planned in the following stages of the research.

#### 2.2.4 Clinically aligned and locally relevant content

The content in *FamNucleus* was curated with a structured, meticulous process to secure clinical and contextual applicability, and usability. The in-app suggestions were informed with national standards of care, while the evidence-based recommendations were optimized based on formative research user-centric feedback. The development team also reviewed and equalized various sources of information to ensure relevant, comprehensive, and clinically proven in-app resources during the early parenting period. These references include Singapore Dietary Guidelines (18) by Health Promotion Board (HPB), Singapore Guidelines for Feeding and Eating in Infants and Young Children (19), guidelines on postnatal care provided by the Ministry of Health, HPB and Families for Life (FFL) parenting resources, and KKH infant-care advisories.

#### 2.2.5 Whole-family design and shared caregiving architecture

*FamNucleus* adopts a whole-family health strategy, allowing parents to tie individual records together as a family. This design indicates mutual dependence between the health behaviors of parents and children, holding joint responsibility in supporting healthy practices in the household. This app aims to align with the real-world dynamics of caregiving by allowing multiple caregivers to engage with the same child profile, which will reinforce the family-centered care principle. This common architecture supports information continuity among caregivers and facilitates coordinated engagement with health-related trackers, education, and goal setting.

#### 2.2.6 Key app features and functional architecture

**Figure 3:**
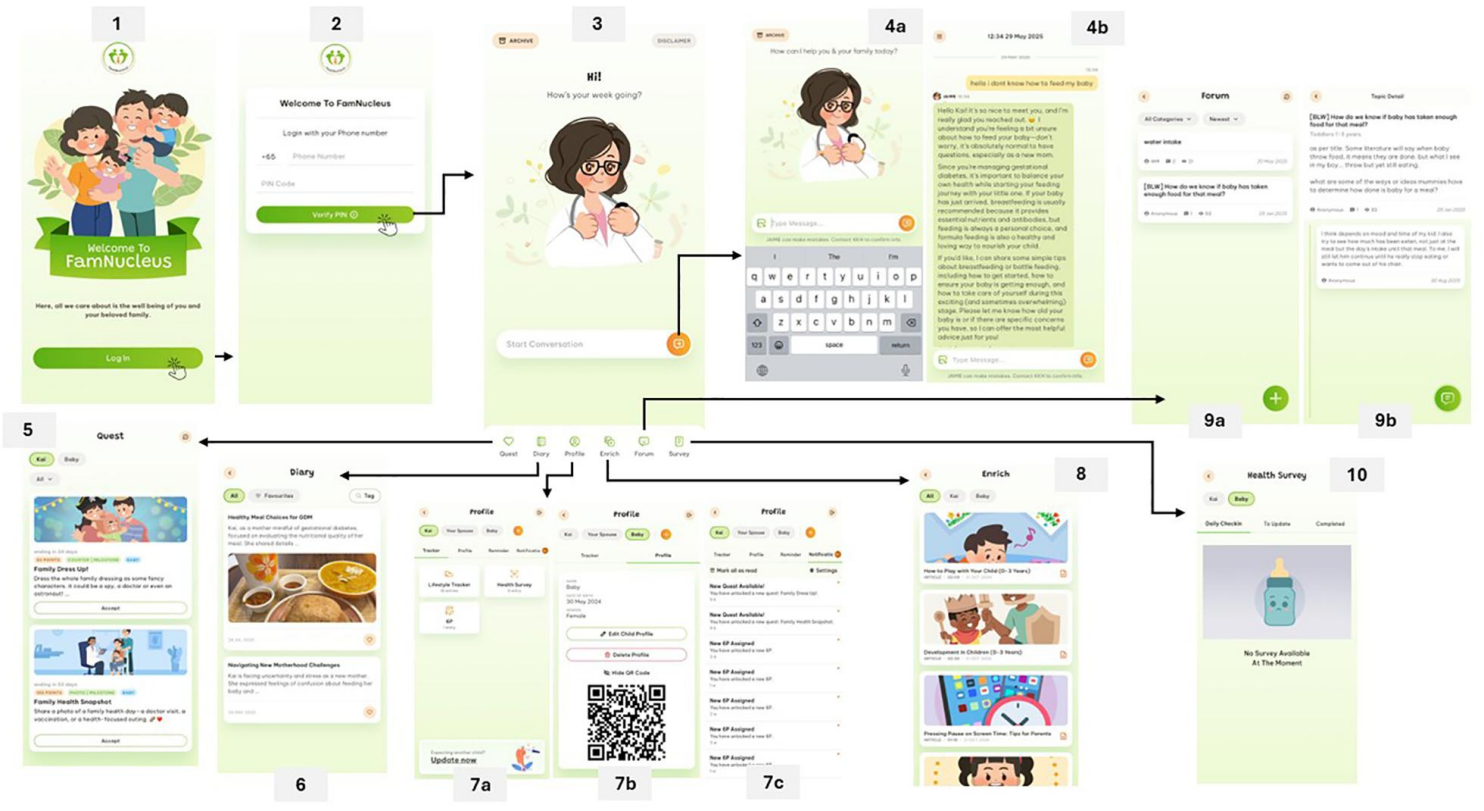
The screenshots are referring to the following user interface: (1) App welcome screen introducing *famNucleus* and its family-centred focus objective. (2) User login and verification screen, enabling secure access via phone number and pin code authentication. (3) Home page with option to start conversation with AI Chatbot (JAIME), initiating user interaction through conversational support. (4a) AI chatbot consultation interface, providing evidence-based, personalized responses to parenting-related queries. (4b) Example of conversation with the AI chatbot. (5) Quest feature that offers interactive, task-based activities to encourage engagement and behavior change. (6) Diary feature that records summary of conversation with JAIME to enable quick renavigation of information. (7a–7c) User profiles and management interfaces, including child profile setup, account management, and QR-code-based access across parents’ account. The Tracker feature can be assessed under Profile, which included lifestyle and 6P monitoring. (8) Enrich feature that delivers curated educational content and parenting resources. (9a–9b) Forum navigation and posting functions, supporting topic exploration and moderated content creation among the users. (10) Health survey interface, designed to collect health-related information and support monitoring when available.

Various important interactive features are emphasized in *FamNucleus* to encourage user engagement and behavioral change. Figure 3 shows the *FamNucleus* user interface and its core features: “Profile” was designed as a personalized user dashboard, while the Health Score Dashboard aggregates profiles of family members into a composite health score to visualize the health trajectories, which corresponds to the national strategy in Singapore to promote early-life health interventions, thereby minimizing the risk of non-communicable diseases. The JAIME (Joint Artificial Intelligence for MEtabolic health) is an AI chatbot layered on the ChatGPT-4.0 Large Language Model, trained using an evidence-informed approach grounded in clinical guidelines and validated educational materials curated by a multidisciplinary team of HCPs to respond to parenting-related queries with evidence-based responses. To reduce the risk of misinformation, JAIME's knowledge base was restricted to pre-reviewed content, and responses were designed to align with established clinical recommendations. To mitigate potential risks of hallucinations, JAIME's outputs were constrained to curated domain-specific content, with iterative review and refinement by clinical experts during development. Users were informed about the role of JAIME as a supportive educational tool which cannot replace professional medical advice. Data protection measures were implemented through secure data handling protocols and compliance with institutional data governance requirements. The chat history with JAIME was then saved under “Diary” for easy retrieval and future references. The “Enrich” includes educational materials and content on parenting, while the “Forum” was developed to facilitate communication among the users. A series of gamification elements were used to increase user engagement, as gamified health education interventions have been shown to potentially improve learning and behavioral outcomes (20, 21). “Quest” was therefore created for parents to complete routine-based quests to gain points or achievement badges. Moreover, “Tracker” was created to enable a stage-specific lifestyle monitoring and logs. The “Survey” component incorporates the 6P nutrition assessment and intervention tool developed under the Healthy Early Life Moments in Singapore (HELMS) program (22), a holistic initiative to promote family health across preconception, pregnancy, and early parenthood. Six core lifestyle domains were assessed using the 6P-tool (23) to monitor parental lifestyle. Lastly, “Nudges” enables push notifications on goal setting, progress tracking, and reminders.

Unlike many existing parenting mHealth apps that primarily provide one-way educational content, *FamNucleus* integrates co-designed bidirectional engagement between parents and HCPs within a real-world clinical workflow. The AI-supported chatbot was trained using evidence-based content developed by a multidisciplinary medical team, ensuring alignment with established clinical guidelines and prior local cohort evidence. In addition, *FamNucleus* includes a shared family profile accessed via QR code, enabling seamless linkage between caregivers and HCPs within a unified digital system. This supports continuity of engagement and coordinated care, which is not commonly available in existing parenting mHealth platforms. This integrated approach of continuous access to evidence-based information, AI-supported guidance, and parent-HCP engagement throughout early caregiving, may facilitate future integration into real-world healthcare delivery models that support parents beyond clinical settings. Collectively, these features position *FamNucleus* as a mHealth digital health intervention.

#### 2.2.7 Clinician-facing dashboard and clinical support oversight

Besides the parent-facing functionalities, *FamNucleus* offers a clinician-facing dashboard, where HCPs can access to aggregated user data analytics, as well as the family profiles. The dashboard was developed to facilitate professional oversight, enhance understanding of family health behaviors between visits, and explore the feasibility of integrating app-generated data into usual clinical practices.

### 2.3 Feasibility **t**esting

The *FamNucleus* MVP feasibility testing employed a convergent parallel mixed-methods study design to include both quantitative and qualitative data collection within the same study timeframe. We used purposive sampling strategy to recruit parents and HCPs in Singapore, with intention to capture perspectives from key target users. As this study was designed as a feasibility and usability evaluation rather than a hypothesis-testing trial, formal sample size calculation was not performed. The sample size for this feasibility testing was guided by established usability testing conventions and pragmatic considerations, whereby a small purposive sample was employed to identify usability issues, inform iterative refinement, assess preliminary acceptability and achieve preliminary thematic saturation in qualitative feedback. Parents were eligible if they were proficient in English with access to a smartphone, and had at least one child aged between 0 and 24 months whom they were actively caring for. Meanwhile, six HCPs with involvement in early childhood care in KKH were also recruited, including two general paediatricians, one dietician, one psychologist, one physiotherapist, and one staff nurse. Ethical approval was obtained from the SingHealth Centralized Institutional Review Board (CIRB Ref: CIRB 2024/2067). Written informed consent was taken from all participants before the data collection.

The participants were onboarded through a study briefing session, consent process, and guided introduction to the *FamNucleus* app. Participants downloaded *FamNucleus* via Android or Apple iOS app stores, and used the app independently for one month (December 2024 to January 2025), during which they were invited to explore all available features. This study was guided by feasibility framework by Bowen et al. (24), which is widely used in early-stage intervention research to evaluate acceptability, demand, implementation, practicality, integration, and limited-efficacy testing prior to a trial. *A priori* feasibility indicators were defined prior to guide evaluation, including (i) recruitment feasibility, defined as successful enrolment of the target sample within the study period; (ii) retention feasibility, defined as completion of follow-up assessments by at least 70% of enrolled participants; (iii) usability and acceptability, defined as achieving at least neutral-to-positive ratings on validated UEQ-KPI and MAUQ scales. Participants first evaluated the *FamNucleus* prototype in December 2024 to assess anticipatory usability, acceptability, and perceived value of the app. They subsequently used the MVP for one month before completing the post-use evaluations in January 2025. Specifically, HCPs were also requested to assess the clinical relevance of the app, content accuracy, and the possibility of its integration into the routine healthcare processes.

#### 2.3.1 Quantitative data collection

To evaluate the usability, acceptability, and satisfaction of *FamNucleus*, participants were asked to fill in two evaluation forms (Supplementary material B): the prototype evaluation form was administered during onboarding to determine users’ first impression of the app, while the MVP evaluation form assessed the user experience after interacting with the app. Cronbach's alpha was used to evaluate the internal consistency of the questionnaires, and the values of 0.70 or above were deemed acceptable (25). The prototype evaluation form was self-administered using a printed questionnaire, which consisted of the modified User Experience Questionnaire-Key Performance Indicator (UEQ-KPI) (26) to assess perceived importance of key user experience dimensions and user version of the Mobile App Rating Scale (uMARS) (27) to evaluate the overall app quality. The UEQ-KPI consisted of six questions on users’ perceived importance of UEQ scale, including perspicuity, efficiency, dependability, stimulation, novelty, and overall attractiveness (26). The UEQ-KPI was rated on a 7-point Likert scale ranging from 1(Not important at all) to 7 (very important) (28). The KPI values were interpreted based on the validated UEQ benchmark framework (29). Meanwhile, the validated uMARS (20 items) was organized into four objective subscales, namely the engagement, functionality, aesthetics, and information, along with a subjective quality (27). All items are rated on a 5-point Likert scale (1 = inadequate to 5 = excellent). Mean scores were calculated for each subscale and overall quality.

After using *FamNucleus* for one month, parents completed the MVP evaluation form in hard copy, while HCPs completed and returned the form electronically via email. The MVP evaluation form consists of UEQ (30), UEQ-KPI (26), and uMARS (27), to assess the user experience, perceived importance of key user experiences dimensions, and overall app quality. The UEQ (30) consists of 26 items to assess the user experience based on pragmatic qualities of the application (perspicuity, efficiency, dependability), hedonic qualities (stimulation, novelty), and overall attractiveness. UEQ was rated on a 7-point semantic differential scale (–3 to +3; where −3 represents the most negative answer, 0 as neutral, and +3 represents the most positive answer). Mean scores above 0.8 indicate a positive evaluation, between −0.8 and 0.8 as a neutral evaluation, and less than −0.8 indicate a negative evaluation (28). Internal consistency of the UEQ assessed using Cronbach's alpha was 0.92 and 0.94 for parents and HCPs, respectively. In addition to the uMARS described earlier, parents also completed additional questions on the perceived impact subscale adapted from uMARS to evaluate the potential influence of this app on their health-related knowledge, attitudes, and behaviors. Cronbach's alpha values for the subscales in uMARS for the overall questionnaire for parents and HCPs were 0.87 and 0.81, respectively. The HCPs completed additional five items adapted from the mHealth App Usability Questionnaire (MAUQ) provider version (31) to assess the clinical relevance and usability of this app in healthcare settings. All HCPs were required to rate the items rated on a 5-point Likert scale (1 = not at all to 5 = highly). Cronbach's alpha value of MAUQ for HCPs was 0.934.

Lastly, parents and HCPs evaluated the clarity and relevance of the content in *FamNucleus*, using a 4-point Likert scale across four criteria: relevance, clarity, simplicity, and ambiguity. The scores ranged from 1 (not relevant/ clear/ simple/ doubtful) to 4 (very relevant/ clear/ simple/ meaning is clear). The Content Validity Index (CVI) was then calculated at the item level (I-CVI) to quantify expert agreement (32). This approach ensures that the intervention content was not only clinically appropriate based on HCPs with expertise in maternal and child health. but also understandable, relevant, and meaningful to the intended end-users like parents. An I-CVI of 0.83 and above were considered acceptable (32).

#### 2.3.2 Qualitative data collection

Following the quantitative surveys, participants were invited to provide feedback through focus group discussions (FGDs) in February 2025. The FGDs explored user experiences, perceived benefits, and suggestions for improvement. The topics of FGDs were divided into five sections: (i) warm-up and context setting, (ii) understanding expectations and initial impressions, (iii) exploring feature and nudge deep-dive, (iv) app engagement, and (v) conclusion. A total of nine FGDs were conducted with four to six parents or two HCPs per group, each lasting about 90 min, with English as the language of communication. The FGDs with parents were conducted in person, with one interviewer and one note-taker present at each session. The FGDs with HCPs were conducted remotely using the Zoom platform, with one interviewer in attendance. The interview guide and questions developed for this study are as shown in Supplementary material C.

Other than gathering responses through open ended questions, a card sorting exercise was incorporated to assess feature value and usability. Participants ranked key features (Quest; Diary/ AI Chatbot; Enrich – including videos, articles, infographics, events, helplines, and nudges; Forum; Survey/Tracker) within a 2 × 2 matrix template (most to least valuable; easiest to most difficult to use). All FGDs were recorded and transcribed verbatim.

### 2.4 Data analysis

Quantitative data were analyzed using IBM SPSS Statistics version 29 (IBM Corp, Armonk, NY, USA). Normality of the data was assessed using the Shapiro–Wilk test, supplemented by visual inspection of histograms. Descriptive statistics were reported as mean ± standard deviation (SD) for normally distributed variables. As UEQ-KPI domain scores were not normally distributed, data were summarized using median, and changes between baseline (T0) and 1-month post-use (T1) were analyzed using the Wilcoxon signed-rank test. Median change (T1−T0) and corresponding 95% confidence intervals (95% CI) were estimated to describe the magnitude and precision of differences between time points. Effect sizes were calculated as r = |Z|/√n, where *n* is the number of paired observations, with values interpreted as small (0.1), moderate (0.3), and large (≥ 0.5) effects (33). Statistical significance was defined as two-tailed *p* < 0.05. Given the exploratory nature of the study, formal adjustment procedures for multiple testing were not applied. However, the potential inflation of type I and type II error was acknowledged, and results were interpreted with appropriate caution.

Meanwhile, qualitative data gathered from the open-ended questions during the FGDs were analyzed with NVivo version 20 (QSR International Pty Ltd, Melbourne, Australia) using a six-step thematic analysis framework (34), namely (i) data familiarization, (ii) generating initial codes, (iii) organizing codes into potential themes, (iv) reviewing themes, (v) defining and naming themes, and (vi) writing the report. Recurring themes related to usability, engagement, and suggestions for improvement were identified from the transcripts, thereby complementing the quantitative results. Given the limited and predefined group of participants, all eligible parents and HCPs were invited to participate in the FGDs. All relevant perspectives within this population were captured, and thematic saturation was considered to have been achieved. Integration was then conducted through methodological triangulation during the results interpretation phase, whereby findings from both datasets were compared and synthesized to identify convergence, complementarity, or divergence.

## 3 Results

### 3.1 Participant characteristics

A total of 36 participants were enrolled, including 30 parents and six HCPs. Five parents provided only partial responses due to drop out and were excluded from the final analysis. Hence, the final sample comprised 31 participants (25 parents and six HCPs). Table 1 presents the socio-demographic profile of participants.

**Table 1:** Baseline socio-demographic and characteristics of participants (*N* = 31**).**

Variables	Parents (*N* = 25)		Healthcare professionals (*N* = 6)	
	Frequency, *n* (%)	Mean ± SD	Frequency, *n* (%)	Mean ± SD
Age	-	31.1 ± 5.4	-	40.2 ± 9.3
Sex Male Female	9 (36.0) 16 (64.0)		2 (33.3) 4 (66.7)	**-**
Monthly household income^a^	-	7,563.2 ± 3,704.0	**-**	15,166.7 ± 6,462.7
Ethnic race Chinese Malay Indian Others	18 (72.0) 7 (28.0) **-** **-**		4 (66.7) 1 (16.7) - 1 (16.7)	
Highest attained education Primary School Secondary (GCE O/N level) ITE/NITEC GCE A level/ Polytechnic/ Diploma University (Bachelors, Masters, PhD)	- 2 (8.0) 3 (12.0) 9 (36.0) 11 (44.0)		- - - 1 (16.7) 5 (83.3)	**-**
Occupation Legislators/ Senior Officials/ Managers Professionals Clerical support workers Service and sales workers Production craftsman Students Police Staff Nurse	2 (8.0) 13 (52.0) 5 (20.0) 2 (8.0) 1 (4.0) 1 (4.0) 2 (8.0) -		- 5 (83.3) - - - - - 1 (16.7)	
Body mass index Underweight Normal Overweight Obese	1 (4.0) 9 (36.0) 8 (32.0) 7 (28.0)	29.9 ± 4.8	- 3 (50.0) 2 (33.3) 1 (16.7)	25.1 ± 5.3

a Currency reported in monthly household income: Singapore Dollar.

Majority of the participants were females (64% for parents; 67% for HCPs), Chinese (72% for parents; 67% for HCPs), and had received tertiary education (44% for parents; 83% for HCPs). All participants have more than 10 years of experience using mobile devices. Among parents, the mean household income was SGD 7563.2 (SD = 3704.0). The mean number of children was 2.1 (SD = 0.7), and the mean age of the youngest child was 6.5 months (SD = 3.0). Among HCPs, the mean duration of professional practice was 16.0 ± 7.7 years.

### 3.2 Usability and acceptability findings

#### 3.2.1 User experience evaluation

The UEQ-KPI was administered at two time points: (i) during the prototype evaluation and (ii) after one month of MVP usage. As shown in Table 2, among parents, a statistically significant decline was observed in perspicuity from prototype to MVP evaluation (median change = −2.00, 95% CI: −2.02 to −0.47; Z = −2.66, r = 0.53; *p* = 0.008), indicating a large effect size. no statistically significant changes were observed in attractiveness, efficiency, dependability, stimulation, and novelty. Among HCPs, a statistically significant decline was observed in perspicuity (median change = −1.00, 95% CI: −1.66 to −0.34; Z = −2.12, r = 0.87; *p* = 0.034), indicating a large effect size. Similarly, no statistically significant changes were observed in attractiveness, efficiency, dependability, stimulation, and novelty. The UEQ-KPI scores during MVP evaluation were within the average benchmark for parents and HCPs, with a mean of 1.01 [SD = 0.86, 95% CI (0.68, 1.34)] and 1.64 [SD = 0.80, 95% CI (1.00, 2.28)], respectively.

**Table 2:** Comparison of median change and its effect size of UEQ-KPI based on domains at baseline and after 1 month of usage.

Domains	Parents (*N* = 25)				HCPs (*N* = 6)			
	Median change (95% CI)	*Z*	*r*	*p* value	Median change (95% CI)	*Z*	*r*	*p* value
Attractiveness	0.00 (−1.21, 0.25)	−1.27	0.25	0.203	0.00 (−1.97, 1.64)	−0.32	0.13	0.748
Perspicuity	−2.00 (−2.02, −0.47)	−2.66	0.53	0.008	−1.00 (−1.66, −0.34)	−2.12	0.87	0.034
Efficiency	−1.00 (−1.24, 0.12)	−1.56	0.31	0.119	−1.00 (−1.94, −0.06)	−1.86	0.76	0.063
Dependability	0.00 (−1.03, −0.43)	−1.27	0.25	0.206	0.00 (−0.87, 1.20)	−0.45	0.18	0.655
Stimulation	0.00 (−1.07, 0.35)	−0.96	0.19	0.337	0.00 (−0.60, 0.26)	−1.00	0.41	0.317
Novelty	−1.00 (−1.21, 0.57)	−0.64	0.13	0.521	−1.00 (−0.38, 1.38)	−1.34	0.55	0.180

T_0_ = Median change from prototype evaluation to MVP evaluation; r = effect size.

**Figure 4:**
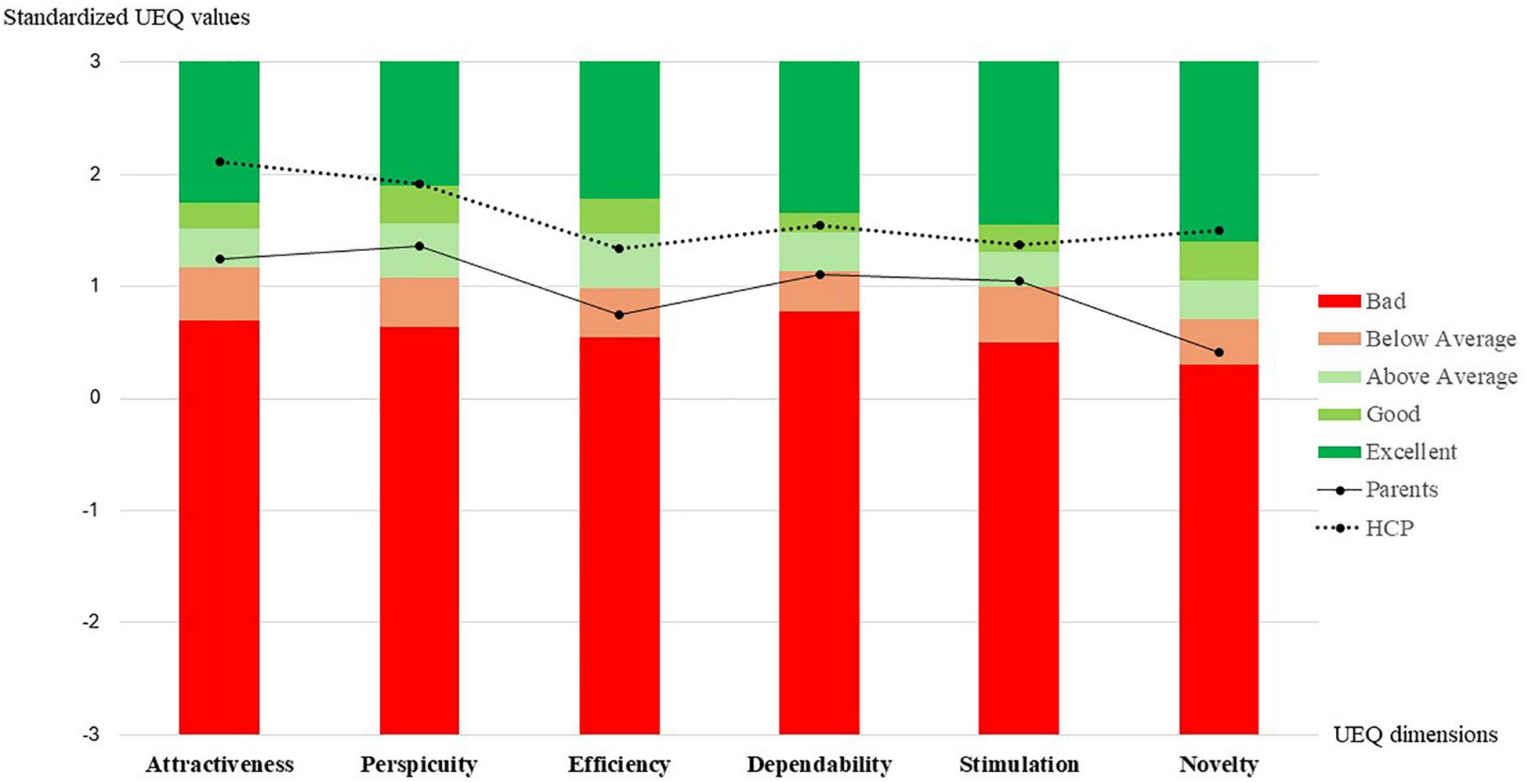
The bar length represents the distribution of scores within each fixed scoring range for the respective dimension. The overlaid line indicates the mean UEQ score for parents and HCPs. The scores are standardized UEQ values ranging from −3 (very negative) to +3 (very positive) (Schrepp *et al*., 2023).

As shown in Figure 4 across all six UEQ subscales for MVP evaluation, parents reported that the attractiveness (1.25 ± 0.97), perspicuity (1.36 ± 0.92), and stimulation (1.05 ± 1.16) was above average, while efficiency (0.75 ± 0.97), dependability (1.11 ± 1.11), and novelty (0.41 ± 1.01) was below average. In contrast, HCPs provided consistently higher ratings compared to parents. Three of the subscales rated by the HCPs fall within the excellent range, namely the attractiveness (2.11 ± 0.75), perspicuity (1.92 ± 0.94), and novelty (1.5 ± 1.14), the dependability (1.54 ± 0.70) and stimulation (1.38 ± 0.54) fall within the good range. The HCPs reported the efficiency (1.33 ± 1.31) of the app as above average. The mean values for each item of the UEQ, along with the SD and CI are reported in the Supplementary material D.

#### 3.2.2 App quality evaluation

The overall app quality mean score rated by the parents and HCPs were 3.99 ± 0.40 and 4.07 ± 0.37, respectively. The mean values of each subscale are displayed in Table 3a, while the mean score of each individual item is shown in Supplementary material E.

Among parents, the information domain received the highest score rating (4.19 ± 0.42), while the lowest was the subjective quality of the app (3.18 ± 0.42). Among the HCPs, the aesthetics domain which comprised three items evaluating the app's layout, graphics, and overall visual appeal, had received the highest score rating (4.39 ± 0.49). Most participants (64% of parents; 83.3% of HCPs) indicated willingness to recommend this app. When asked about potential future use of this app, about 40% of the parents perceived that they will use it moderately for 3–5 times, while half of the HCPs (50%) indicated they would have frequent app usage (10–50 times) over the next 12 months. Majority of the parents had low willingness to pay for the app (76%). Over half of the participants (52% of parents; 83.3% of HCPs) gave positive rating for *FamNucleus*.

#### 3.2.3 App usability evaluation

As shown in Table 3b, half of the HCPs (50%) highly agreed that this app provided an acceptable way to deliver healthcare services. All of the HCPs (100%) agreed that this app would be useful for healthcare practice and can improve the access for healthcare service delivery. In terms of patient management, more than half of the HCPs (66.7%) indicated that the app helps manage patients’ health effectively. Additionally, 83.3% rated the app as an acceptable tool for delivering healthcare services such as accessing educational materials, tracking patients’ activities, and self-assessment. The overall mean MAUQ score was 4.23 ± 0.61, indicating a generally positive usability and perceived usefulness for HCPs.

**Table 3:** Mean, standard deviation, and range values of app quality evaluation based on uMARS, and healthcare professionals' evaluation of clinical relevance and usability based on MAUQ.

(a) Mean, standard deviation, and range values of app quality evaluation based on uMARS.				
Subscales	Parents (*N* = 25)		HCPs (*N* = 6)	
	Mean ± SD	Range	Mean ± SD	Range
Section A: Engagement	3.59 ± 0.68	2.20–4.60	3.63 ± 0.56	3.00–4.40
Section B: Functionality	4.07 ± 0.44	3.25–4.50	4.04 ± 0.40	3.50–4.50
Section C: Aesthetics	4.12 ± 0.60	2.67–5.00	4.39 ± 0.49	3.67–5.00
Section D: Information	4.19 ± 0.42	3.00–4.75	4.21 ± 0.37	3.75–4.75
Section E: Subjective quality	3.18 ± 0.54	2.00–4.00	3.58 ± 0.63	2.75–4.50
Section F: Perceived impact	3.60 ± 0.59	2.33–4.83	-	-

(b) HCPs’ evaluation of clinical relevance and usability of the app based on mHealth App Usability Questionnaire (MAUQ).					
Items	Score distribution, *n* (%)				
	Not at all	Slightly	Neutral	Agree	Highly agree
The app provides an acceptable way to deliver health care services.	0 (0)	0 (0)	1 (16.7)	2 (33.3)	3 (50.0)
The app will be useful for health care practice.	0 (0)	0 (0)	0 (0)	3 (50.0)	3 (50.0)
The app improves access to delivering health care services.	0 (0)	0 (0)	0 (0)	4 (66.6)	2 (33.3)
The app helps to manage patients’ health effectively	0 (0)	0 (0)	2 (33.3)	3 (50.0)	1 (16.7)
The app provides an acceptable way to deliver health care services, such as accessing educational materials, tracking patients’ activities and self-assessment.	0 (0)	0 (0)	1 (16.7)	3 (50.0)	2 (33.3)

HCPs, Healthcare professionals; SD, Standard deviation.

### 3.3 Content validation

Among parents, the I-CVI values were 0.92 for relevance, 0.96 for clarity, 0.96 for simplicity, and 0.80 for ambiguity. Among HCPs, I-CVI values were 1.00 for relevance, 1.00 for clarity, 1.00 for simplicity, and 0.83 for ambiguity, demonstrating strong agreement on content quality. While content validity was positive across most domains, both parents and HCPs highlighted minor concerns related to ambiguity.

### 3.4 Thematic findings from qualitative feedback

**Figure 5:**
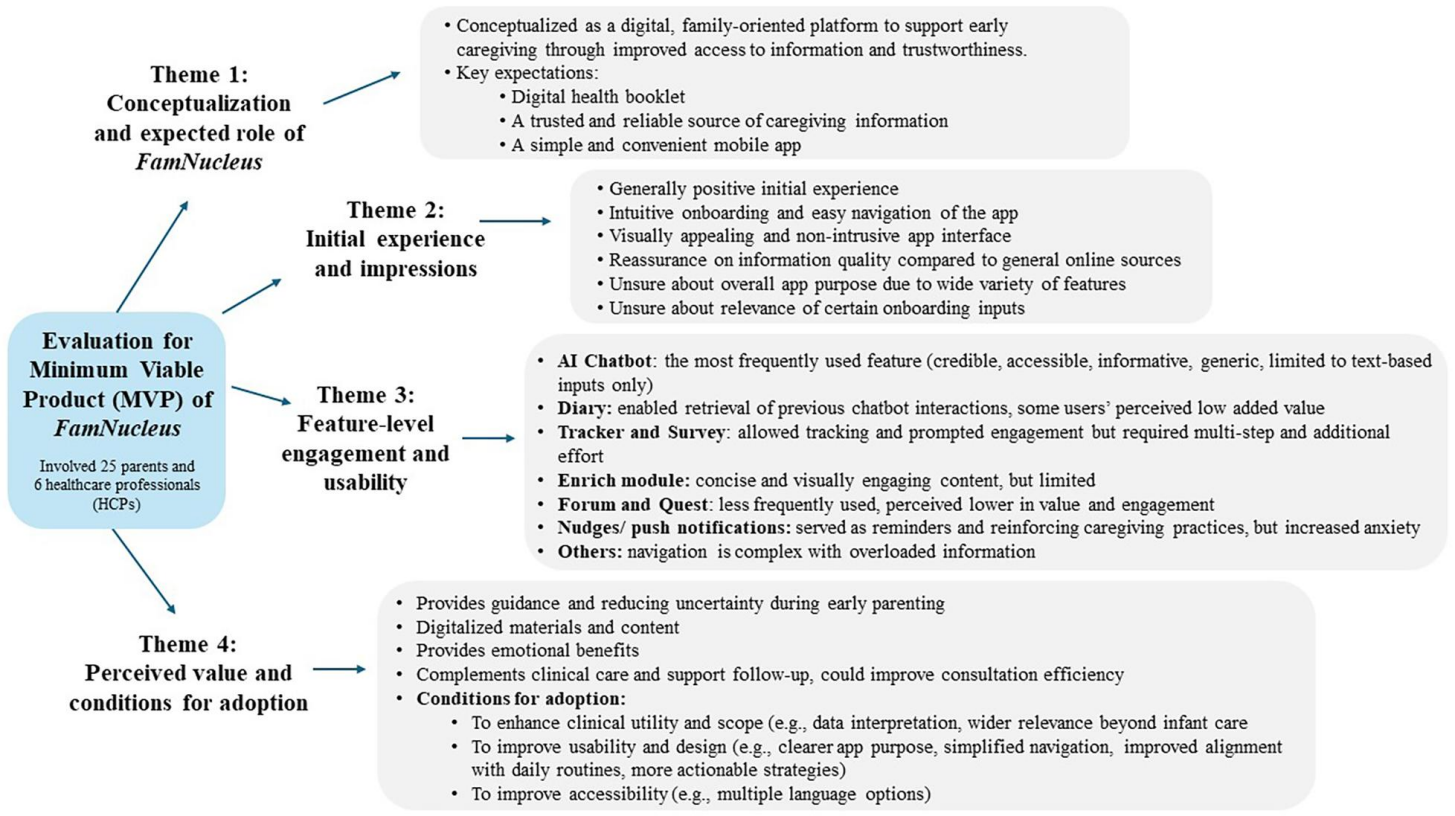
Mind map of themes and subthemes identified from focus group discussions exploring participants' experiences, perceptions, and recommendations related to FamNucleus minimum viable product.

Four themes were identified based on the progression from conceptualization to post-use evaluation (Figure 5).

#### 3.4.1 Theme 1: Conceptualization and expected role of famNucleus

Participants conceptualized *FamNucleus* as a digital, family-focused platform that supports early caregiving through improved access to information and credibility. One of the key expectations was that *FamNucleus* could be used as a digital health booklet, allowing convenient storing and retrieving information on child health:

“I was expecting something also fairly simple. But at the same time, digitalized which is easy for us to track.. I think having this would be convenient for us to refer to. We won't be home whenever they ask these questions. When we are at home also, we are busy with the baby. But then, the book will be home. We got no time to flip through and all that. So, actually digital is convenient.” – FGD_P5

The participants anticipated the app to be a trusted and reliable provider of caregiving resources, offering a point of reference to minimize the need to rely on general online search engine.

“Currently, we are heavily utilising Google. It would be great if there is something for me to reference to. At least, the material inside is being filtered in a sense.” – FGD_P2

“Online, there’s a lot of information which is visible but I don’t know if I can trust them. But if it is on this app, then I am okay.” – FGD_P3

In general, participants anticipated *FamNucleus* to be a simple, convenient, and trustworthy digital mHealth resource for caregiving support (Table 4).

**Table 4:** Theme 1: conceptualization and expected role of *FamNucleus.*

Conceptual expectation	Description
Digital health booklet	Expected to function as a digital alternative to the physical health booklet, enabling convenient storage and retrieval of child health information
Trusted and reliable information source	Expected to provide credible, clinically trustworthy information compared with general online sources
Single reference point	Expected to consolidate caregiving information into one platform to reduce reliance on multiple sources
Convenience and accessibility	Expected to improve access to caregiving information anytime and across contexts

#### 3.4.2 Theme 2: Initial experience and impressions

The first impressions with *FamNucleus* were mostly favorable, as participants reported positive onboarding process, stating it as an intuitive process with easy navigation within the app. The majority of users could explore the app independently without requiring formal instruction. The app user interface was reported to be visually appealing and non-intrusive, contributing to a favorable first impression.

“The illustrations are very cute. They do pull you in. I like that. I think overall, as an app, it really tries to get you to interact.” – FGD_HCP3

“There is no popup that just comes screaming in your face. There is something that you have to do. There is something that I want you to see. It is just calm. It is just ‘Hello, how can I help you?” – FGD_P1

Participants shared that they were reassured when accessing health-related resources on *FamNucleus*, especially when compared with information from other general online sources.

“My first thought on this is like, this should have been the thing… very convenient. A lot of questions back then would have been better answered by this than if we Google… It is just that sense of security as a parent. Really parenting is really scary. It is scary enough. Google just made it scarier.”- FGD_P1

Nevertheless, there were challenges in understanding the overall purpose of *FamNucleus* and how its components fit together.

“Currently now I open the app, and I have so many things that are on the app. And I don’t really know the main purpose of it.” – FGD_F3

Some participants also raised questions on the relevance of some data inputs during onboarding, specifically those not directly relevant to child care.

“I think the parents’ details are unnecessary. Parents being parents, we are always more concerned about our little ones. For us ourselves, I think we are old enough to manage our own.” – FGD_P2

In general, usability and design of *FamNucleus* were positively evaluated and well received. However, the clarity of app purpose and coherence of the system remained as the key gaps in early user experience (Table 5).

**Table 5:** Theme 2: initial experience and impressions.

Aspect	Experience from participants
Onboarding and navigation	Simple, intuitive, and easy to follow; users could explore independently; some onboarding inputs (e.g., parent details) perceived as unnecessary
User interface and design	Visually appealing, engaging, and non-intrusive; absence of disruptive pop-ups
Emotional response	Provided reassurance compared with uncertainty from general online sources
System coherence	Multiple functions available within one platform; difficulty understanding how different components should be used together; lack of clarity regarding overall purpose and how features fit together

#### 3.4.3 Theme 3: Feature-level engagement and usability

Participants engaged differentially with app, with variation in perceived usefulness and usability across features:

The AI Chatbot “JAIME” was the most frequently used feature and was perceived as a credible and accessible source of information that provided practical caregiving support.

“The Chatbot, can ask anything. And I find that it is more trustable compared to google. Because this is by doctors. Google is literally anybody can just put out information.” – FGD_P1

“They were just giving me quite a lot of routines, adjust a bit timing. And maybe do a bathe before that. Give the child a dark environment. It will help the child to maybe start sleeping earlier time. So, it does give me practical steps.” – FGD_P3

However, responses were sometimes perceived as generic and limited to text-based input.

“I think it was more generic answer. It always prompts you to the right answer which is to go and seek professional advice.” – FGD_P2

“Apart from accepting text-based input, it will be good if it accepts images. Let’s say you take a picture of the poo and you can just upload it and it will maybe analyse on the backend and let you know, is it normal or are you required to seek medical attention to look at the thing.” – FGD_P4

The Diary feature enabled retrieval of previous chatbot interactions, although some participants questioned its added value.

“Diary is useful to go back and refer to. Because they do tag the conversation as well. And what the information that they have provided for us. So that we have the exact text history.” – FGD_P1

“Let’s say I want to ask the same question again; I can always do it back at the chatbot. So, I don’t really see a need to review the diary, to read back what I actually asked.” – FGD_P4

The Tracker and Survey features supported awareness through tracking and prompted engagement, although usability was limited by multi-step input and effort required.

“I’ve never tracked anything unless it was medically required, but with the app’s reminders, I started tracking my child’s food. I realized things I wouldn’t have otherwise—like on certain days, she eats more, and on others, less. This kind of tracking is very beneficial.” – FGD_HCP1

“For the survey tracker, you have to press in. Press more. It is not really user friendly. You really spend a lot of time going in and press.” – FGD_P2

The Enrich module was positively received for its concise and visually engaging content, although some participants preferred more efficient formats.

“The one that I like most was actually Enrich because it actually gave some information in a quite nice graphics and it is all short enough for us to just catch a quick video to learn more about how to care for your infant.” – FGD_P3

“I didn’t have the time to do it. I personally prefer to read. If you are reading, you can skim through. You can read fast. But if 26 s, means you have to watch 26 s of the video.” – FGD_P1

Some features, such as Forum and Quest, were less used and viewed as less valuable, with engagement limited by moderation delays and unclear incentives.

“When you post it in, you have to wait for administrator to approve it. And then, if somebody comments on it, you still have to wait for the administrator to approve comment which I wasn’t really interested in that function.” – FGD_P3

“It is quite easy to accept the quest but then it is quite hard for parents to commit to the quest. Like, there’s no benefits when completing the quest. We get points but that’s all. Maybe we are wishing for voucher maybe for the kids or what.” – FGD_P2

Participants shared that the nudges and push notifications from *FamNucleus* reminded them and reinforced their caregiving practices. Nevertheless, some participants stated that they experienced anxiety from these nudges.

“That was useful. It even corrects some of the things that I am currently doing to make it better… It gives information that is valuable.” – FGD_P3

“…they’re helpful, especially for new parents. They serve as reminders and reinforce good habits.” – FGD_HCP5

“I think the nudges can be helpful to some extent and can also create more anxiety sometimes.” – FGD_HCP3

Across features, participants highlighted barriers for long-term usage, including the complexity to navigate within *FamNucleus*, as well as overwhelming in-app information.

“This app already has everything that the other app has, just that it is too much information. It is good. But just that there are too many things to click on instead of a simplified one.” – FGD_P2

**Figure 6:**
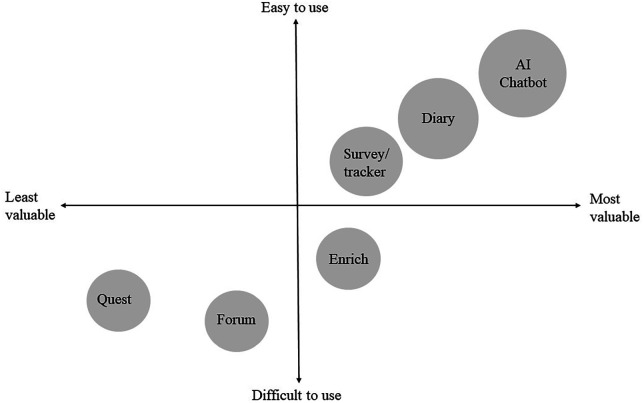
Bubble sizes reflect the frequency of mentions by the parents.

**Figure 7:**
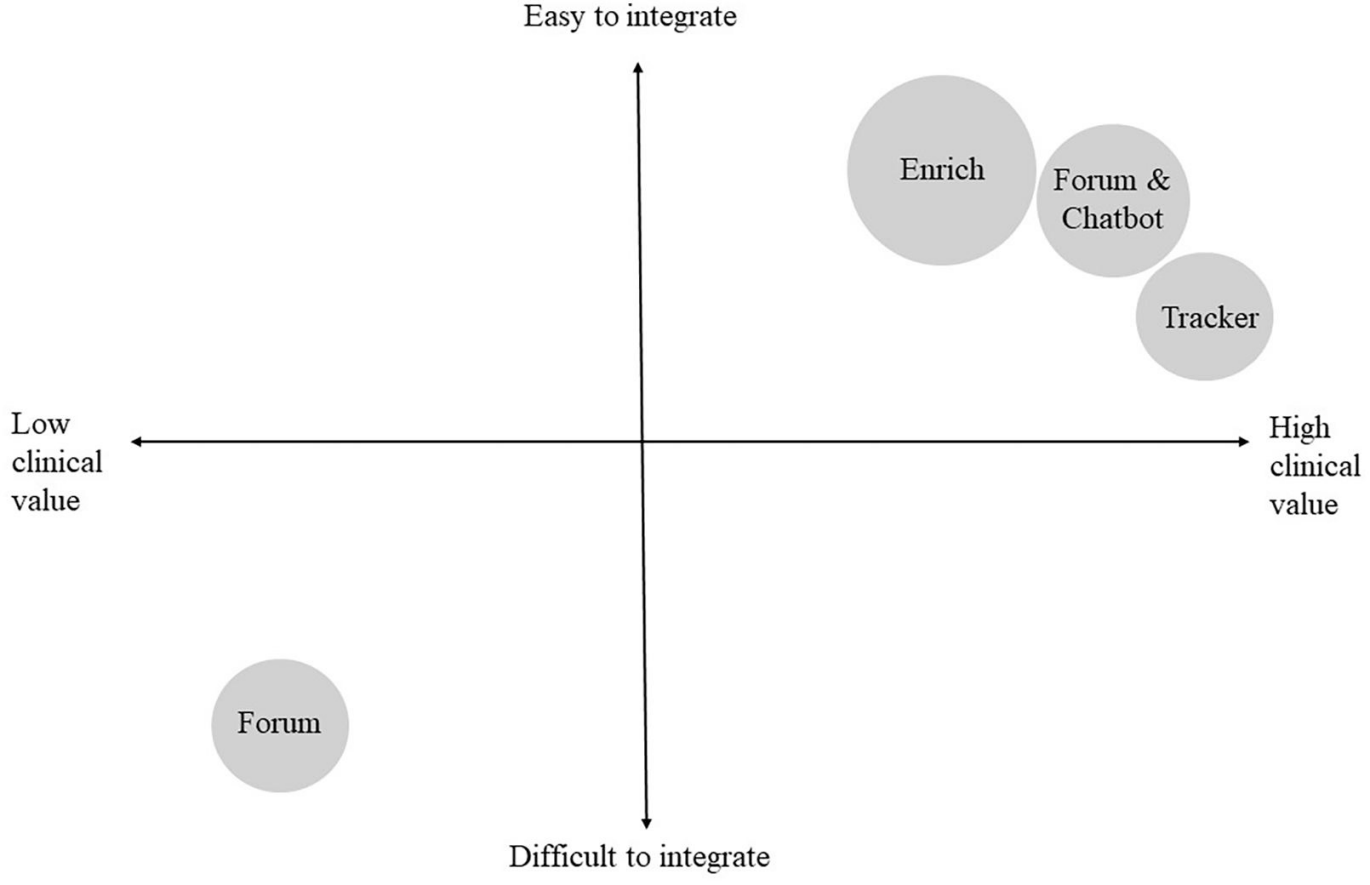
Bubble sizes reflect the frequency of mentions by the HCPs.

Consistent findings have been reported in the pattern of feature prioritization among users. For instance, the AI Chatbot, Diary, and Tracker were perceived as features with higher values in *FamNucleus*, while Forum and Quest seemed as less valuable and more inconvenient to use (Figures 6, 7). Table 6 outlines an overview of user interactions with each feature of *FamNucleus*.

**Table 6:** Theme 3: feature-level engagement and usability.

Feature	What worked well	What didn't work
AI Chatbot	Credible, accessible source of information; provided practical caregiving support	Responses sometimes generic; limited to text-based input
Diary	Enabled retrieval of previous chatbot interactions through stored history	Limited perceived added value
Survey/Tracker	Supported awareness through tracking; prompted engagement	Multi-step, time-consuming input; high effort required
Enrich	Concise, visually engaging educational content; useful for quick learning	Less efficient for users preferring text; limited depth for some needs
Forum	Potential for peer interaction and shared experiences	Moderation delays; low responsiveness; limited engagement
Quest	Encouraged interaction through tasks	Low perceived value; lack of incentives; difficult to sustain engagement
Nudges/notifications	Provided reminders and reinforced caregiving practices	Context-dependent usefulness; could increase anxiety
Overall usability	Multiple functions available within one platform	Navigation complexity; information overload; too many steps

#### 3.4.4 Theme 4: Perceived value and conditions for adoption

Among parents, *FamNucleus* was greatly appreciated and valued for offering real-time guidance and reducing uncertainty during early parenting.

“My child is only two years old, so I’m still quite new to parenting… When you read online, there’s just so much information, and it’s hard to know what to trust. But with an app like this, new parents can log and track what their child is doing and receive guided advice.” – FGD_HCP1

Some participants also highlighted the convenience of parenting tool in digital format, as well as gaining emotional benefits from the app usage.

“It is more convenient as compared with bringing the book. It is digitalized and you and your partner can use. In terms of emergency outside when you are not at home. You don’t just bring the health book with you all the time.” – FGD_P1

“I had blues so they actually help me how to calm down myself and stuff like that.” – FGD_P5

Among the HCPs, *FamNucleus* was viewed as a digital tool that may complement clinical care and support follow-up visits during postpartum care.

“It complements. Currently, resources are scattered—this brings them together. If implemented well, it bridges the gap between clinic visits, supports home-based care and reinforces what’s discussed in sessions. I think it’s a strong start, and with a few refinements, it could be a valuable clinical companion.” – HCP_P5

“In my work with community families, having accessible videos and articles helps reinforce discussions. It supports follow-up between consultations.” – FGD_HCP5

HCPs highlighted that *FamNucleus* could enhance the efficiency of consultation through less repetitive clarifications and improving parents’ understanding outside of clinics. The integration of *FamNucleus* into routine clinical care would be based on alignment with clinical practice and formal endorsement as a reliable digital tool. The HCPs also highlighted opportunities to enhance the app's clinical utility and scope, particularly by improving data interpretation and expanding its relevance beyond infant care.

“Let’s say it can be monthly basis or quarterly basis. Comes out with a statistic to show you what are all the foods the baby has tried let’s say in the first quarter or the first month. It is able to compile the data and give a statistic kind of thing.” – HCP_P4

“Normally, when we go for a doctor’s check, what will happen, they might tell you, ‘Your baby is not gaining weight.’ Then, it is a red flag there. Or maybe it suddenly drops. Or if let’s say there is another way to record intake there. Or the reason for the drop there. Could it be because of too much of activities? Is there a cause to cut down the activities. Maybe have a bit more purpose in the tracking.” – HCP_P4

“I think a lot of mommies, after we have trouble to go back in shape as well, that will be also a useful idea I think, that mommy also can use it for calories on their own food as well.” – HCP_P4

On the user level, sustained adoption was conditional on app usability and design, including clearer app purpose, simplified navigation, and better customization to align with daily caregiving routines, as well as more actionable content and enhanced searchability (Table 7). Lastly, some users suggested that the app accessibility may be enhanced by having multiple language options to accommodate diverse user groups.

**Table 7:** Theme 4: perceived value and conditions for adoption.

Domain	What contributed to value	What limited value/required refinement
Support for parents	Provided guidance, reduced uncertainty, supported caregiving decisions; emotional reassurance	Value dependent on relevance to user needs
Convenience and accessibility	Digital access to child information; reduced reliance on physical booklet; shared caregiver access	Limited integration with healthcare systems
Clinical integration (HCP perspective)	Reinforced clinical advice; supported follow-up; improved consultation efficiency	Requires alignment with clinical practice and formal endorsement
Clinical utility and scope (HCP perspective)	Potential to support decision-making through data insights; extend to maternal health	Limited data interpretation features; current scope focused mainly on infant care
Usability and adoption	Integrated multiple functions within one platform	Need for clearer purpose, simplified navigation, and better alignment with caregiving routines
Accessibility	Potential to support diverse user groups	Need for multi-language support

### 3.5 Integration of quantitative and qualitative findings

To strengthen the integration of findings from the mixed-methods design, a convergence matrix (Table 8) was developed to synthesize quantitative results with corresponding qualitative themes, highlighting the areas of convergence and explanatory insight across both sets of data. Several quantitative findings were strongly supported by qualitative data. In general, *FamNucleus* was perceived as a promising and valuable digital health tool that supports caregiving and extensive clinical care. Lower perspicuity scores observed during the MVP evaluation were consistent with qualitative reports of initial confusion regarding app navigation and uncertainty about the app purpose. Aesthetic quality of FamNucleus was consistently supported across quantitative and qualitative data. Similarly, small declines in perceived efficiency and novelty were corroborated by participants’ descriptions on reasons for disengagement and perceived low values for app features like Forum and Quest. While overall app quality was acceptable based on quantitative results, the qualitative data provide a more comprehensive understanding of user experience, highlighting opportunities to enhance long-term engagement. Overall, feasibility targets were achieved. Recruitment was completed within the planned study period, with a retention rate of 86.1% exceeding the predefined threshold for feasibility. Usability and acceptability outcomes indicated generally positive user experiences, as reflected in UEQ-KPI and MAUQ scores above neutral benchmarks.

**Table 8:** Joint display of quantitative and qualitative findings.

Quantitative result summary	Qualitative themes	Triangulation of findings
Lower perspicuity score observed at MVP phase compared with prototype	Theme 1: Conceptualization and expected role of *FamNucleus* Theme 2: Initial experience and impressions	Convergence: Quantitative decrease in perspicuity aligns with qualitative responses on early usability confusion, indicating the need for improved onboarding and guidance.
Positive aesthetics ratings across users	Theme 2: Initial experience and impressions	Complementarity: Aesthetic quality consistently supported across quantitative and qualitative data.
Small declines in perceived efficiency and novelty among parents over time	Theme 3: Feature-level engagement and usability Theme 4: Perceived value and conditions for adoption	Complementarity**:** Quantitative lower scores explained by qualitative feedback on reasons for disengagement and perceived low values.
HCPs rated generally positive score on clinical usability	Theme 4: Perceived value and conditions for adoption	Convergence: Strong agreement between perceived functional performance and positive usability ratings by the HCPs.
Overall acceptable perceived app quality	Theme 4: Perceived value and conditions for adoption	Complementarity: Overall positive perception supported, but qualitative data highlights opportunities to enhance long-term engagement.

## 4 Discussion

This mixed-method study presents a comprehensive evaluation of the *FamNucleus* mHealth app as the quantitative usability metrics are triangulated with qualitative user responses. Parents and HCPs from diverse backgrounds were involved in the evaluation, which added a wide spectrum of perspectives to the evaluation. Nevertheless, most participants in current study were highly digitally literate, which may have contributed to more favorable usability and acceptability ratings, as experienced users are generally more adept at navigating digital interfaces and adapting to app-based workflows. In contrast, usability challenges may be more pronounced among individuals with lower digital literacy, or non-English-speaking users, where difficulties in navigation, comprehension, and sustained engagement may reduce perceived ease of use and overall acceptability.

In general, *FamNucleus* was well-accepted by both parents and HCPs, indicating its potential as a family-centered digital health platform. Nevertheless, the findings on usability were mixed. Significant declines in perspicuity and efficiency were observed in the post-use evaluation, indicating a gap between initial impressions and sustainable usability. While initial app exposure may capture novelty-driven ease of use, sustained app interaction appears to have revealed underlying challenges. For instance, parents may have increased cognitive burden in real-world caregiving and clinical contexts, as users were required to switch attention between daily tasks and app navigation (35). In addition, the presence of fragmented app features, such as Quest and Forum, may have confused the participants on how components relate to one another and lead to reduced perspicuity as users progress beyond initial exploration. Furthermore, limited onboarding depth may have resulted in shallow initial learning, making the app less intuitive over time when participants attempted more complex or repeated interactions.

Parents often reported lack of confidence in infant care practices, particularly on feeding and introduction of food. The strong preference for locally relevant and clinically credible information supports the importance of positioning *FamNucleus* as a reliable, context-specific digital health resource, which aligns with evidence that has been shown to be culturally grounded in early caregiving in Singapore (7, 36). The content of *FamNucleus* is consistent with national initiatives, such as the Healthier SG (37) and Grow Well SG (38), further supporting its relevance in the local healthcare context. Diverse expectations of *FamNucleus* were observed, in which HCPs consistently rated the app more favorably than parents. HHCPs will tend to focus more on clinical plausibility and possible real-world applicability, while parents might focus on ease of use, immediacy and applicability of the app. The higher perceived utility among HCPs could be attributed by the availability of a clinician-facing dashboard. This dissimilarity highlights the complexity of designing clinical strength and the usability of the interface by general users, especially in dual-user interface platforms (39). The perception of reduced importance in prototype and MVP is no exception compared to the other studies in the field of digital health, likely explained by the usability restrictions that occurred in the real-world practice (40).

The perception of *FamNucleus* was seen as both aesthetically attractive and meaningful as indicated by positive ratings on the attractiveness and the stimulation scales. One of the perceived key strengths included its ability to consolidate fragmented resources to a single platform. In the meantime, triangulated results suggest that reduced perspicuity and efficiency score may be attributed to uncertain app positioning, disjointed navigation, and augmented cognitive load caused by various features. *FamNucleus* obtained low score for novelty because many parents were unsure of the main purpose of this app even though there is a wide variety of in-app features. Parental tracking and shared family records were some key features that were underutilized. These discoveries underscore the importance of a clear purpose and information architecture in early app engagement. Feature-level patterns support this interpretation. The AI Chatbot, Diary, and Tracker were highly valued as they offer immediate actionable support to parents, while Forum and Quest were not well engaged due to low perceived relevance, inefficient and slow moderation process, and lack of incentives. Limited Forum activity also reflects insufficient network effects within a small user base. Collectively, engagement seems to be motivated by the perceived utility and convenience more than feature availability. From a behavioral perspective, these usability issues can be interpreted as opportunity constraints of the COM-B model, such that structural and environmental barriers may limit effective interactions. For example, challenges related to navigation, time constraints, and caregiving demands reflected limitations in physical and social opportunity, while feedback on content clarity and ease of understanding related to psychological capability. Participants’ perceived usefulness and willingness to continue using the app also suggested reflective motivation to engage with the intervention. Together, these qualitative findings provide preliminary behavioral insights aligned with the COM-B framework and highlight areas for refinement prior to larger-scale effectiveness evaluation. Addressing these limitations may improve sustained use and promote behavior change.

HCPs emphasized the potential of *FamNucleus* to supplement clinical workflows with the reinforcement of consultation recommendations and continuity of care. The clinician-backed credibility of *FamNucleus* and the ability to consolidate of fragmented resources were the main strengths. Prior research revealed that digital interventions endorsed by clinicians are linked to higher trust, engagement, satisfaction, and health outcomes (41–44). Nonetheless, an effective implementation will require integration into healthcare systems, such as clinician endorsement to approve it as a primary resource that is compatible with the current clinical workflows. Enhancing data interpretation and generating actionable summaries may further strengthen clinical utility.

## 5 Strengths and limitations

The mixed-methods study design allowed robust triangulation of usability measures in terms of user experience, which provided both objective and contextual insights. The multidisciplinary team co-development process maximized clinical relevance and cultural suitability, whereas the presence of both parents and HCPs provided both user and implementation perspectives. Nevertheless, there are several limitations to be taken into consideration. The sample size of current study was relatively small, predominantly female, Chinese, highly educated, English-speaking, and digitally literate. While this enhances internal consistency, it may have restricted the generalizability as higher educational attainment and digital literacy have been associated with more favorable perceptions of mHealth usability and acceptance in prior research. Consequently, the usability and acceptability ratings observed in this study may represent a more optimistic assessment compared to those expected in more socioeconomically, linguistically, and digitally diverse populations. Nevertheless, this demographic profile likely reflects the primary target user population of *FamNucleus*, where digital health adoption, English literacy, and smartphone use are generally high among younger parents seeking healthcare information and support. As such, feedback from this user group remains valuable for informing iterative refinement of the app functionality. The small sample size may also limit statistical inference and increase the risk of Type II error. The short evaluation period precludes assessment of long-term engagement and objective behavioral changes. User experience might have been affected by technical problems during MVP testing. This study did not apply formal adjustments for multiple comparisons across domains, which may increase the risk of Type I error. The content validity assessment was conducted with a relatively small panel of experts and end-users, which may limit the stability and generalizability of the CVI estimates. Hence, findings should therefore be viewed as preliminary, some statistically significant findings should be interpreted with caution. Future studies with larger sample sizes should consider confirmatory analyses with appropriate control for multiple testing. There was no overlap between the development and evaluation teams, which reduced the risk of evaluator bias due to intervention familiarity. This risk was partially reduced through independent data collection and analysis conducted by researchers who were not involved in the app development, although residual bias in study design, implementation, and interpretation cannot be fully excluded. The study did not include objective usage data, such as app logs or completion rates to assess app engagement level. The interpretations of engagement and feature utilization were based on self-reported and qualitative data only. This limits conclusions regarding actual usage behavior, and future studies should incorporate objective analytics to validate perceived engagement. Future hybrid effectiveness-implementation studies are warranted to determine the long-term parent-child health outcomes, including parental self-efficacy, emotional well-being, and outcomes related to child growth and development. Other implementation barriers and facilitators should be identified, including cost-effectiveness and sustainability.

### 5.1 Recommendations for refinement and future efficacy testing

Ongoing efforts are underway for the app refinement based on the feedback and implementation insights acquired from the current pilot study. These refinements included optimizing the onboarding and app positioning to facilitate better understanding of the purpose of *FamNucleus*, enhancing integration of app functionalities and navigation paths, strengthening personalized nudges and actionable suggestions based on structured approaches and guidance, which include employing the 6P-tool (23), Breastfeeding Education and Screening Tool (45), and Feeding, Lifestyle, Activity Goals (46). These refinements must focus on purpose clarity, ease of navigation, and minimizing cognitive load instead of increasing feature complexity. These measures may reinforce the role of *FamNucleus* as an effective and practical decision-support mHealth parenting tool. As this study was limited to short-term feasibility testing and user evaluation, real-world implementation outcomes were not formally assessed. While current findings suggested feasibility and HCPs’ willingness to integrate the application into clinical workflows, scaling to institutional or national levels may introduce additional workflow burden. Large-scale deployment would require sustained maintenance of the AI system and content database, as well as consideration of long-term operational costs. Therefore, future studies should evaluate implementation strategies in larger and more diverse populations over longer follow-up periods (e.g., across 12–18 months of the early caregiving journey) that balance scalability with workflow efficiency, sustainability, and cost-effectiveness. In addition, intervention trials to assess effectiveness may help evaluate both clinical outcomes and implementation-related factors, including workflow burden, long-term maintenance of AI-supported infrastructure, and sustainability within real-world healthcare settings.

## 6 Conclusion

*FamNucleus* demonstrates preliminary feasibility and acceptability as a family-centered mHealth app. Both parents and HCPs recognized its potential to help in early caregiving and complementing clinical care. *FamNucleus* addresses key gaps in infant, maternal, and family health through a combination of behavioral science, clinical expertise, and user-centered app design. Mixed usability findings were observed, including reduced perspicuity over time, uncertainty regarding app purpose, and limited engagement with certain features such as forums and gamification. These findings highlight ongoing usability and navigation challenges that may affect sustained engagement. Following additional refinement by addressing usability barriers associated with app clarity, navigation, and feature integration, future studies should further evaluate sustainability, behavioral outcomes, and effectiveness of real-world implementation.

## Data Availability

The raw data supporting the conclusions of this article will be made available by the authors, without undue reservation.
