# Supplementary material for: Development, feasibility testing and evaluation of a family-oriented mobile application to promote healthy lifestyle in infants and parents during early life: a mixed methods study
Source: Front Digit Health. 2026 Jun 19;8:1869210. doi: 10.3389/fdgth.2026.1869210 (PMC13329725; doi:10.3389/fdgth.2026.1869210)
Supplement: Supplementary file 1 [file Datasheet1.docx]

Supplementary Material

| Supplementary Material A | Mapping intervention techniques with Capability, Opportunity, Motivation-Behavior (COM-B) theoretical domains in *FamNucleus*. |
| --- | --- |
| Supplementary Material B | Prototype and MVP evaluation forms |
| Supplementary Material C | *FamNucleus* User Evaluations: Focus Group Discussion (FGD) Guide |
| Supplementary Material D | Detailed evaluations of the UEQ for MVP phase |
| Supplementary Material E | Mean, standard deviation (SD), minimum and maximum values for the scales and subscales of the Mobile Application Rating Scale: User Version (uMARS) |

# Supplementary Material A:

# Table 1. Mapping intervention techniques with Capability, Opportunity, Motivation-Behavior (COM-B) theoretical domains in *FamNucleus*.

| **Features/ Conceptual app functionalities** | **Description** | **COM-B Domains (Theoretical Basis)** | |
| --- | --- | --- | --- |
| Profile | This feature allows parents to create and personalize their account, tailoring content to family context. Using a QR code, parents can link multiple users to form a family profile, ensuring that app content and progress tracking are shared appropriately across family members. | Capability | Knowledge |
|  |  | Opportunity | Self-identity; personalization; social/ professional role and identity |
| AI Chatbot (JAIME) | An in-app trained AI-Chatbot named JAIME (Joint Artificial Intelligence for MEtabolic health) aims to provide interactive, personalized conversations to support users in managing postpartum physical recovery, health, mental wellness, diet, physical activity, mood, and parenting. | Capability | Information; skills; resources; emotion |
|  |  | Opportunity | Resources |
|  |  | Motivation | Feedback; reassurance |
| Diary | This feature serves to log conversations with JAIME by automatically storing the conversations. The tagging and “favourite” function enable users to easily retrieve and revisit important information for future reference. | Capability | Information; skills; attention and decision processes; awareness |
|  |  | Opportunity | Reinforcement |
|  |  | Motivation | Behavioral regulation |
| Enrich | This feature provides age- and role-specific educational content for parents. | Capability | Knowledge |
|  |  | Opportunity | Environmental context and resources |
|  |  | Motivation | Beliefs about capabilities and consequences |
| Forum | This feature enables peer-to-peer sharing, discussion, and social support within the app. Users can observe the actions and outcomes of others’ behaviors, exchange ideas on topics of interest, and receive encouragement from peers. | Opportunity | Social influences |
| Quest | Weekly quests to encourage user engagement and adoption of healthy behaviors. | Opportunity | Associative learning; reinforcement |
|  |  | Motivation | Goal-setting; reinforcement; rewards |
| Tracker | The tracker feature allows parents to log and track lifestyle indices, reflections, experiences, or child-related notes, alongside tools to track food intake, weight, and activity. This feature enhances self-monitoring and behavioral regulation, supporting parents’ capability to observe patterns and evaluate progress toward their goals. | Capability | Attention and decision processes; self-monitoring |
|  |  | Motivation | Behavioral regulation |
| Survey | This feature allows users to complete self-assessments for up to 24 months postpartum and receive personalized feedback to support improvements in health and wellbeing. | Capability | Attention and decision processes |
|  |  | Opportunity | Social role and identity |
|  |  | Motivation | Goals setting; intentions; beliefs about capabilities and consequences; behavioral regulation |
| Nudges and push notifications on goal-setting, progress tracking, reminders. | Users will receive structured bite-sized content and nudges to support sustained behavior change. The content is provided in different formats to improve familiarity and strengthen the users’ memory. | Capability | Knowledge; skills; memory |
|  |  | Opportunity | Environmental context and resources; reinforcement |
|  |  | Motivation | Optimism; intentions; goals; behavioral regulation |
| Dashboard | A dashboard feature was developed exclusively for healthcare professionals (HCPs), enabling them to access the overall dashboard and view family profiles, with results retrieved from the back-end system. | Capability | Attention and decision processes |

**Supplementary Material B:**

# Form 1: Prototype evaluation form (Parents)

Participant Serial No: _____________ Date of data collection (dd/mm/yyyy): _____________

**Rate the importance of these factors for your app user experience. All items are rated on a 7-point scale from “1. Not important at all” to “7. Very Important”.**

|  |  | 1 | 2 | 3 | 4 | 5 | 6 | 7 |
| --- | --- | --- | --- | --- | --- | --- | --- | --- |
| 1 | The product should look attractive, enjoyable, friendly and pleasant. | Ο | Ο | Ο | Ο | Ο | Ο | Ο |
| 2 | I should perform my tasks with the product fast, efficient and in a pragmatic way. | Ο | Ο | Ο | Ο | Ο | Ο | Ο |
| 3 | The product should be easy to understand, clear, simple, and easy to learn. | Ο | Ο | Ο | Ο | Ο | Ο | Ο |
| 4 | The interaction with the product should be predictable, secure and meets my expectations. | Ο | Ο | Ο | Ο | Ο | Ο | Ο |
| 5 | Using the product should be interesting, exciting and motivating. | Ο | Ο | Ο | Ο | Ο | Ο | Ο |
| 6 | The product should be innovative, inventive and creatively designed. | Ο | Ο | Ο | Ο | Ο | Ο | Ο |

**Select the number that most accurately represents the quality of the app you are rating. All items are rated on a 5-point scale from “1.Inadequate” to “5.Excellent”.**

1. **Does the app look fun/entertaining to use?**

| 1  Not at all | 2 | 3 | 4 | 5  Highly |
| --- | --- | --- | --- | --- |

1. **Does the app look interesting to use? Does it present its information in an interesting way compared to other similar apps?**

| 1  Not at all | 2 | 3 | 4 | 5  Highly |
| --- | --- | --- | --- | --- |

1. **Does the app allow you to customise the settings and preferences that you would like such as fonts, contents, and notifications?**

| 1  Not at all | 2 | 3 | 4 | 5  Highly |
| --- | --- | --- | --- | --- |

1. **Does the app allow user input, provide feedback, and contain prompts such as reminders, sharing options, notifications, etc.?**

| 1  Not at all | 2 | 3 | 4 | 5  Highly |
| --- | --- | --- | --- | --- |

1. **Does the app content (visuals, language, design) look appropriate for the target audience?**

| 1  Not at all | 2 | 3 | 4 | 5  Highly |
| --- | --- | --- | --- | --- |

1. **Do the arrangement and size of buttons, icons, menus, and content on the screen appear appropriate?**

| 1  Not at all | 2 | 3 | 4 | 5  Highly |
| --- | --- | --- | --- | --- |

1. **How high is the quality/resolution of graphics used for buttons, icons, menus, and content?**

| 1  Very poor | 2 | 3 | 4 | 5  Very good |  |
| --- | --- | --- | --- | --- | --- |

1. **How appealing is the app's appearance?**

| 1  Ugly | 2 | 3 | 4 | 5  Beautiful |  |
| --- | --- | --- | --- | --- | --- |

1. **Will the app be useful for you and your family health and well-being?**

| 1  Not at all | 2 | 3 | 4 | 5  Highly |
| --- | --- | --- | --- | --- |

1. **What is your overall (star) rating of the app?**

| 1 *  One of the worst apps I’ve seen | 2 ** | 3 *** | 4 **** | 5 *****  One of the best apps I’ve seen |
| --- | --- | --- | --- | --- |

| **Further comments about the app?** |
| --- |

# Form 2: Prototype evaluation form (Healthcare professionals)

Participant Serial No: _____________ Date of Data Collection (dd/mm/yyyy): _____________

**Rate the importance of these factors for your app user experience. All items are rated on a 7-point scale from “1. Not important at all” to “7. Very Important”.**

|  |  | 1 | 2 | 3 | 4 | 5 | 6 | 7 |
| --- | --- | --- | --- | --- | --- | --- | --- | --- |
| 1 | The product should look attractive, enjoyable, friendly and pleasant. | Ο | Ο | Ο | Ο | Ο | Ο | Ο |
| 2 | I should perform my tasks with the product fast, efficient and in a pragmatic way. | Ο | Ο | Ο | Ο | Ο | Ο | Ο |
| 3 | The product should be easy to understand, clear, simple, and easy to learn. | Ο | Ο | Ο | Ο | Ο | Ο | Ο |
| 4 | The interaction with the product should be predictable, secure and meets my expectations. | Ο | Ο | Ο | Ο | Ο | Ο | Ο |
| 5 | Using the product should be interesting, exciting and motivating. | Ο | Ο | Ο | Ο | Ο | Ο | Ο |
| 6 | The product should be innovative, inventive and creatively designed. | Ο | Ο | Ο | Ο | Ο | Ο | Ο |

**Select the number that most accurately represents the quality of the app you are rating. All items are rated on a 5-point scale from “1.Inadequate” to “5.Excellent”.**

1. **Does the app look fun/entertaining to use?**

| 1  Not at all | 2 | 3 | 4 | 5  Highly |
| --- | --- | --- | --- | --- |

1. **Does the app look interesting to use? Does it present its information in an interesting way compared to other similar apps?**

| 1  Not at all | 2 | 3 | 4 | 5  Highly |
| --- | --- | --- | --- | --- |

1. **Does the app allow you to customise the settings and preferences that you would like such as fonts, contents, and notifications?**

| 1  Not at all | 2 | 3 | 4 | 5  Highly |
| --- | --- | --- | --- | --- |

1. **Does the app allow user input, provide feedback, and contain prompts such as reminders, sharing options, notifications, etc.?**

| 1  Not at all | 2 | 3 | 4 | 5  Highly |
| --- | --- | --- | --- | --- |

1. **Does the app content (visuals, language, design) look appropriate for the target audience?**

| 1  Not at all | 2 | 3 | 4 | 5  Highly |
| --- | --- | --- | --- | --- |

1. **Do the arrangement and size of buttons, icons, menus, and content on the screen appear appropriate?**

| 1  Not at all | 2 | 3 | 4 | 5  Highly |
| --- | --- | --- | --- | --- |

1. **How high is the quality/resolution of graphics used for buttons, icons, menus, and content?**

| 1  Very poor | 2 | 3 | 4 | 5  Very good |  |
| --- | --- | --- | --- | --- | --- |

1. **How appealing is the app's appearance?**

| 1  Ugly | 2 | 3 | 4 | 5  Beautiful |  |
| --- | --- | --- | --- | --- | --- |

1. **Will the app provide an acceptable way to deliver health care services?**

| 1  Not at all | 2 | 3 | 4 | 5  Highly |
| --- | --- | --- | --- | --- |

1. **Will the app be useful for health care practice?**

| 1  Not at all | 2 | 3 | 4 | 5  Highly |
| --- | --- | --- | --- | --- |

1. **Will the app improve access to delivering health care services?**

| 1  Not at all | 2 | 3 | 4 | 5  Highly |
| --- | --- | --- | --- | --- |

1. **Will the app help to manage patients’ health more effectively?**

| 1  Not at all | 2 | 3 | 4 | 5  Highly |
| --- | --- | --- | --- | --- |

1. **Will the app provide an acceptable way to deliver health care services, such as accessing educational materials, tracking patients’ activities and self-assessment?**

| 1  Not at all | 2 | 3 | 4 | 5  Highly |
| --- | --- | --- | --- | --- |

1. **What is your overall (star) rating of the app?**

| 1 *  One of the worst apps I’ve seen | 2 ** | 3 *** | 4 **** | 5 *****  One of the best apps I’ve seen |
| --- | --- | --- | --- | --- |

| **Further comments about the app?** |
| --- |

# Form 3: MVP evaluation form (Parents)

Participant Serial No: _____________ Date of Data Collection (dd/mm/yyyy): _____________

**Section A: The questionnaire consists of pairs of contrasting attributes that may apply to the app. The circles between the attributes represent gradations between the opposites. You can express your agreement with the attributes by selecting the circle that most closely reflects your impression.**

**Example:**

**
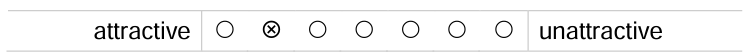
**

**This response would mean that you rate the application as more attractive than unattractive.**

**Please decide spontaneously. Don’t think too long about your decision to make sure that you convey your original impression.**

**Sometimes you may not be completely sure about your agreement with a particular attribute or you may find that the attribute does not apply completely to the particular product. Nevertheless, please tick a circle in every line.**

**It is your personal opinion that counts. Please remember: there is no wrong or right answer!**

**Please assess the app now by selecting one circle per line.**

|  |  | 1 | 2 | 3 | 4 | 5 | 6 | 7 |  |
| --- | --- | --- | --- | --- | --- | --- | --- | --- | --- |
| 1 | Annoying | Ο | Ο | Ο | Ο | Ο | Ο | Ο | Enjoyable |
| 2 | Not understandable | Ο | Ο | Ο | Ο | Ο | Ο | Ο | Understandable |
| 3 | Creative | Ο | Ο | Ο | Ο | Ο | Ο | Ο | Dull |
| 4 | Easy to learn | Ο | Ο | Ο | Ο | Ο | Ο | Ο | Difficult to learn |
| 5 | Valuable | Ο | Ο | Ο | Ο | Ο | Ο | Ο | Inferior |
| 6 | Boring | Ο | Ο | Ο | Ο | Ο | Ο | Ο | Exciting |
| 7 | Not interesting | Ο | Ο | Ο | Ο | Ο | Ο | Ο | Interesting |
| 8 | Unpredictable | Ο | Ο | Ο | Ο | Ο | Ο | Ο | Predictable |
| 9 | Fast | Ο | Ο | Ο | Ο | Ο | Ο | Ο | Slow |
| 10 | Inventive | Ο | Ο | Ο | Ο | Ο | Ο | Ο | Conventional |
| 11 | Obstructive | Ο | Ο | Ο | Ο | Ο | Ο | Ο | Supportive |
| 12 | Good | Ο | Ο | Ο | Ο | Ο | Ο | Ο | Bad |
| 13 | Complicated | Ο | Ο | Ο | Ο | Ο | Ο | Ο | Easy |
| 14 | Unlikable | Ο | Ο | Ο | Ο | Ο | Ο | Ο | Pleasing |
| 15 | Usual | Ο | Ο | Ο | Ο | Ο | Ο | Ο | Leading edge |
| 16 | Unpleasant | Ο | Ο | Ο | Ο | Ο | Ο | Ο | Pleasant |
| 17 | Secure | Ο | Ο | Ο | Ο | Ο | Ο | Ο | Not secure |
| 18 | Motivating | Ο | Ο | Ο | Ο | Ο | Ο | Ο | Demotivating |
| 19 | Meets expectations | Ο | Ο | Ο | Ο | Ο | Ο | Ο | Does not meet expectations |
| 20 | Inefficient | Ο | Ο | Ο | Ο | Ο | Ο | Ο | Efficient |
| 21 | Clear | Ο | Ο | Ο | Ο | Ο | Ο | Ο | Confusing |
| 22 | Impractical | Ο | Ο | Ο | Ο | Ο | Ο | Ο | Practical |
| 23 | Organised | Ο | Ο | Ο | Ο | Ο | Ο | Ο | Cluttered |
| 24 | Attractive | Ο | Ο | Ο | Ο | Ο | Ο | Ο | Unattractive |
| 25 | Friendly | Ο | Ο | Ο | Ο | Ο | Ο | Ο | Unfriendly |
| 26 | Conservative | Ο | Ο | Ο | Ο | Ο | Ο | Ο | Innovative |

**Section B: Rate the importance of these factors for your app user experience. All items are rated on a 7-point scale from “1. Not important at all” to “7. Very Important”.**

|  |  | 1 | 2 | 3 | 4 | 5 | 6 | 7 |
| --- | --- | --- | --- | --- | --- | --- | --- | --- |
| 1 | The product looks attractive, enjoyable, friendly and pleasant. | Ο | Ο | Ο | Ο | Ο | Ο | Ο |
| 2 | I can perform my tasks with the product fast, efficient and in a pragmatic way. The user interface looks organized. | Ο | Ο | Ο | Ο | Ο | Ο | Ο |
| 3 | The product is easy to understand, clear, simple, and easy to learn. | Ο | Ο | Ο | Ο | Ο | Ο | Ο |
| 4 | The interaction with the product is predictable, secure and meets my expectations. | Ο | Ο | Ο | Ο | Ο | Ο | Ο |
| 5 | Using the product is interesting, exciting and motivating. | Ο | Ο | Ο | Ο | Ο | Ο | Ο |
| 6 | The product is innovative, inventive and creatively designed. | Ο | Ο | Ο | Ο | Ο | Ο | Ο |

**Section C: Select the number that most accurately represents the quality of the app you are rating. All items are rated on a 5-point scale from “1. Inadequate” to “5. Excellent”.**

1. **Is the app fun/entertaining to use? Does it have components that make it more fun than other similar apps?**

1 Dull, not fun or entertaining at all

2 Mostly boring

3 OK, fun enough to entertain user for a brief time (< 5 minutes)

4 Moderately fun and entertaining, would entertain user for some time (5-10 minutes total)

5 Highly entertaining and fun, would stimulate repeat use

1. **Is the app interesting to use? Does it present its information in an interesting way compared to other similar apps?**

1 Not interesting at all

2 Mostly uninteresting

3 OK, neither interesting nor uninteresting; would engage user for a brief time (< 5 minutes)

4 Moderately interesting; would engage user for some time (5-10 minutes total)

5 Very interesting, would engage user in repeat use

1. **Does it allow you to customise the settings and preferences that you would like to (e.g. sound, content and notifications)?**

1 Does not allow any customisation or requires setting to be input every time

2 Allows little customisation and that limits app’s functions

3 Basic customisation to function adequately

4 Allows numerous options for customisation

5 Allows complete tailoring the user’s characteristics/preferences, remembers all settings

1. **Does it allow user input, provide feedback, contain prompts (reminders, sharing options, notifications, etc.)?**

1 No interactive features and/or no response to user input

2 Some, but not enough interactive features which limits app’s functions

3 Basic interactive features to function adequately

4 Offers a variety of interactive features, feedback and user input options

5 Very high level of responsiveness through interactive features, feedback and user input options

1. **Is the app content (visuals, language, design) appropriate for the target audience?**

1 Completely inappropriate, unclear or confusing

2 Mostly inappropriate, unclear or confusing

3 Acceptable but not specifically designed for the target audience. May be inappropriate/unclear/confusing at times

4 Designed for the target audience, with minor issues

5 Designed specifically for the target audience, no issues found

1. **How accurately/fast do the app features (functions) and components (buttons/menus) work?**

1 App is broken; no/insufficient/inaccurate response (e.g. crashes/bugs/broken features, etc.)

2 Some functions work, but lagging or contains major technical problems

3 App works overall. Some technical problems need fixing, or is slow at times

4 Mostly functional with minor/negligible problems

5 Perfect/timely response; no technical bugs found, or contains a ‘loading time left’ indicator (if relevant)

1. **How easy is it to learn how to use the app; how clear are the menu labels, icons and instructions?**

1 No/limited instructions; menu labels, icons are confusing; complicated

2 Takes a lot of time or effort

3 Takes some time or effort

4 Easy to learn (or has clear instructions)

5 Able to use app immediately; intuitive; simple (no instructions needed)

1. **Does moving between screens make sense; Does app have all necessary links between screens?**

1 No logical connection between screens at all /navigation is difficult

2 Understandable after a lot of time/effort

3 Understandable after some time/effort

4 Easy to understand/navigate

5 Perfectly logical, easy, clear and intuitive screen flow throughout, and/or has shortcuts

1. **Do taps/swipes/pinches/scrolls make sense? Are they consistent across all components/screens?**

1 Completely inconsistent/confusing

2 Often inconsistent/confusing

3 OK with some inconsistencies/confusing elements

4 Mostly consistent/intuitive with negligible problems

5 Perfectly consistent and intuitive

1. **Is arrangement and size of buttons, icons, menus and content on the screen appropriate?**

1 Very bad design, cluttered, some options impossible to select, locate, see or read

2 Bad design, random, unclear, some options difficult to select/locate/see/read

3 Satisfactory, few problems with selecting/locating/seeing/reading items

4 Mostly clear, able to select/locate/see/read items

5 Professional, simple, clear, orderly, logically organised

1. **How high is the quality/resolution of graphics used for buttons, icons, menus and content?**

1 Graphics appear amateur, very poor visual design - disproportionate, stylistically inconsistent

2 Low quality/low resolution graphics; low quality visual design – disproportionate

3 Moderate quality graphics and visual design (generally consistent in style)

4 High quality/resolution graphics and visual design – mostly proportionate, consistent in style

5 Very high quality/resolution graphics and visual design - proportionate, consistent in style throughout

1. **How good does the app look?**

1 Ugly, unpleasant to look at, poorly designed, clashing, mismatched colours

2 Bad – poorly designed, bad use of colour, visually boring

3 OK – average, neither pleasant, nor unpleasant

4 Pleasant – seamless graphics – consistent and professionally designed

5 Beautiful – very attractive, memorable, stands out; use of colour enhances app features/menus

1. **Is app content correct, well written, and relevant to the goal/topic of the app?**

1 Irrelevant/inappropriate/incoherent/incorrect

2 Poor. Barely relevant/appropriate/coherent/may be incorrect

3 Moderately relevant/appropriate/coherent/and appears correct

4 Relevant/appropriate/coherent/correct

5 Highly relevant, appropriate, coherent, and correct

1. **Is the information within the app comprehensive but concise?**

1 Minimal or overwhelming

2 Insufficient or possibly overwhelming

3 OK but not comprehensive or concise

4 Offers a broad range of information, has some gaps or unnecessary detail; or has no links to more information and resources

5 Comprehensive and concise; contains links to more information and resources

1. **Is visual explanation of concepts – through charts/graphs/images/videos, etc. – clear, logical, correct?**

1 Completely unclear/confusing/wrong or necessary but missing

2 Mostly unclear/confusing/wrong

3 OK but often unclear/confusing/wrong

4 Mostly clear/logical/correct with negligible issues

5 Perfectly clear/logical/correct

1. **Does the information within the app seem to come from a credible source?**

1 Suspicious source

2 Lacks credibility

3 Not suspicious but legitimacy of source is unclear

4 Possibly comes from a legitimate source

5 Definitely comes from a legitimate/specialised source

1. **Would you recommend this app to people who might benefit from it?**

1 Not at all I would not recommend this app to anyone

2 There are very few people I would recommend this app to

3 Maybe There are several people I would recommend this app to

4 There are many people I would recommend this app to

5 Definitely I would recommend this app to everyone

1. **How many times do you think you would use this app in the next 12 months if it was relevant to you?**

| 1  None | 2  1-2 | 3  3-10 | 4  10-50 | 5  >50 |
| --- | --- | --- | --- | --- |

1. **Would you pay for this app?**

| 1  Definitely not | 2 | 3 | 4 | 5  Definitely yes |
| --- | --- | --- | --- | --- |

1. **What is your overall (star) rating of the app?**

| 1 *  One of the worst apps I’ve used | 2 ** | 3 ***  Average | 4 **** | 5 *****  One of the best apps I’ve used |
| --- | --- | --- | --- | --- |

1. **This app has increased my awareness of the importance of addressing the health behaviour.**

| 1  Strongly disagree | 2 | 3 | 4 | 5  Strongly agree |
| --- | --- | --- | --- | --- |

1. **This app has increased my knowledge/understanding of the health behaviour.**

| 1  Strongly disagree | 2 | 3 | 4 | 5  Strongly agree |
| --- | --- | --- | --- | --- |

1. **The app has changed my attitudes toward improving the health behaviour.**

| 1  Strongly disagree | 2 | 3 | 4 | 5  Strongly agree |
| --- | --- | --- | --- | --- |

1. **The app has increased my intentions/motivation to address the health behaviour.**

| 1  Strongly disagree | 2 | 3 | 4 | 5  Strongly agree |
| --- | --- | --- | --- | --- |

1. **This app would encourage me to seek further help to address the health behaviour (if I needed it).**

| 1  Strongly disagree | 2 | 3 | 4 | 5  Strongly agree |
| --- | --- | --- | --- | --- |

1. **Use of this app will increase/decrease the health behaviour in my family.**

| 1  Strongly disagree | 2 | 3 | 4 | 5  Strongly agree |
| --- | --- | --- | --- | --- |

**Section D: Please rate the overall content of the app based on the 4-point scale below. Please tick (√) in the appropriate box based on your rating for the four criteria.**

|  | **Criteria** | **Score** | **Overall rating** |
| --- | --- | --- | --- |
| 1 | Relevance | 1= not relevant |  |
|  |  | 2= items need some revision |  |
|  |  | 3= relevant but need minor revision |  |
|  |  | 4= very relevant |  |
| 2 | Clarity | 1= not clear |  |
|  |  | 2= items need some revision |  |
|  |  | 3= clear but need minor revision |  |
|  |  | 4= very clear |  |
| 3 | Simplicity | 1= not simple |  |
|  |  | 2= items need some revision |  |
|  |  | 3= simple but need minor revision |  |
|  |  | 4= very simple |  |
| 4 | Ambiguity | 1= doubtful |  |
|  |  | 2= items need some revision |  |
|  |  | 3= no doubt but need minor revision |  |
|  |  | 4= meaning is clear |  |

| **Further comments about the app?** |
| --- |

# Form 4: MVP evaluation form (Healthcare professionals)

Participant Serial No: _______ Date of Data Collection (dd/mm/yyyy): _____________

**Section A: The questionnaire consists of pairs of contrasting attributes that may apply to the app. The circles between the attributes represent gradations between the opposites. You can express your agreement with the attributes by selecting the circle that most closely reflects your impression.**

**Example:**

**
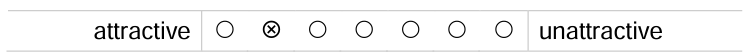
**

**This response would mean that you rate the application as more attractive than unattractive.**

**Please decide spontaneously. Don’t think too long about your decision to make sure that you convey your original impression.**

**Sometimes you may not be completely sure about your agreement with a particular attribute or you may find that the attribute does not apply completely to the particular product. Nevertheless, please tick a circle in every line.**

**It is your personal opinion that counts. Please remember: there is no wrong or right answer!**

**Please assess the app now by selecting one circle per line.**

|  |  | 1 | 2 | 3 | 4 | 5 | 6 | 7 |  |
| --- | --- | --- | --- | --- | --- | --- | --- | --- | --- |
| 1 | Annoying | Ο | Ο | Ο | Ο | Ο | Ο | Ο | Enjoyable |
| 2 | Not understandable | Ο | Ο | Ο | Ο | Ο | Ο | Ο | Understandable |
| 3 | Creative | Ο | Ο | Ο | Ο | Ο | Ο | Ο | Dull |
| 4 | Easy to learn | Ο | Ο | Ο | Ο | Ο | Ο | Ο | Difficult to learn |
| 5 | Valuable | Ο | Ο | Ο | Ο | Ο | Ο | Ο | Inferior |
| 6 | Boring | Ο | Ο | Ο | Ο | Ο | Ο | Ο | Exciting |
| 7 | Not interesting | Ο | Ο | Ο | Ο | Ο | Ο | Ο | Interesting |
| 8 | Unpredictable | Ο | Ο | Ο | Ο | Ο | Ο | Ο | Predictable |
| 9 | Fast | Ο | Ο | Ο | Ο | Ο | Ο | Ο | Slow |
| 10 | Inventive | Ο | Ο | Ο | Ο | Ο | Ο | Ο | Conventional |
| 11 | Obstructive | Ο | Ο | Ο | Ο | Ο | Ο | Ο | Supportive |
| 12 | Good | Ο | Ο | Ο | Ο | Ο | Ο | Ο | Bad |
| 13 | Complicated | Ο | Ο | Ο | Ο | Ο | Ο | Ο | Easy |
| 14 | Unlikable | Ο | Ο | Ο | Ο | Ο | Ο | Ο | Pleasing |
| 15 | Usual | Ο | Ο | Ο | Ο | Ο | Ο | Ο | Leading edge |
| 16 | Unpleasant | Ο | Ο | Ο | Ο | Ο | Ο | Ο | Pleasant |
| 17 | Secure | Ο | Ο | Ο | Ο | Ο | Ο | Ο | Not secure |
| 18 | Motivating | Ο | Ο | Ο | Ο | Ο | Ο | Ο | Demotivating |
| 19 | Meets expectations | Ο | Ο | Ο | Ο | Ο | Ο | Ο | Does not meet expectations |
| 20 | Inefficient | Ο | Ο | Ο | Ο | Ο | Ο | Ο | Efficient |
| 21 | Clear | Ο | Ο | Ο | Ο | Ο | Ο | Ο | Confusing |
| 22 | Impractical | Ο | Ο | Ο | Ο | Ο | Ο | Ο | Practical |
| 23 | Organised | Ο | Ο | Ο | Ο | Ο | Ο | Ο | Cluttered |
| 24 | Attractive | Ο | Ο | Ο | Ο | Ο | Ο | Ο | Unattractive |
| 25 | Friendly | Ο | Ο | Ο | Ο | Ο | Ο | Ο | Unfriendly |
| 26 | Conservative | Ο | Ο | Ο | Ο | Ο | Ο | Ο | Innovative |

**Section B: Rate the importance of these factors for your app user experience. All items are rated on a 7-point scale from “1. Not important at all” to “7. Very Important”.**

|  |  | 1 | 2 | 3 | 4 | 5 | 6 | 7 |
| --- | --- | --- | --- | --- | --- | --- | --- | --- |
| 1 | The product looks attractive, enjoyable, friendly and pleasant. | Ο | Ο | Ο | Ο | Ο | Ο | Ο |
| 2 | I can perform my tasks with the product fast, efficient and in a pragmatic way. The user interface looks organized. | Ο | Ο | Ο | Ο | Ο | Ο | Ο |
| 3 | The product is easy to understand, clear, simple, and easy to learn. | Ο | Ο | Ο | Ο | Ο | Ο | Ο |
| 4 | The interaction with the product is predictable, secure and meets my expectations. | Ο | Ο | Ο | Ο | Ο | Ο | Ο |
| 5 | Using the product is interesting, exciting and motivating. | Ο | Ο | Ο | Ο | Ο | Ο | Ο |
| 6 | The product is innovative, inventive and creatively designed. | Ο | Ο | Ο | Ο | Ο | Ο | Ο |

**Section C: Select the number that most accurately represents the quality of the app you are rating. All items are rated on a 5-point scale from “1. Inadequate” to “5. Excellent”.**

1. **Is the app fun/entertaining to use? Does it have components that make it more fun than other similar apps?**

1 Dull, not fun or entertaining at all

2 Mostly boring

3 OK, fun enough to entertain user for a brief time (< 5 minutes)

4 Moderately fun and entertaining, would entertain user for some time (5-10 minutes total)

5 Highly entertaining and fun, would stimulate repeat use

1. **Is the app interesting to use? Does it present its information in an interesting way compared to other similar apps?**

1 Not interesting at all

2 Mostly uninteresting

3 OK, neither interesting nor uninteresting; would engage user for a brief time (< 5 minutes)

4 Moderately interesting; would engage user for some time (5-10 minutes total)

5 Very interesting, would engage user in repeat use

1. **Does it allow you to customise the settings and preferences that you would like to (e.g. sound, content and notifications)?**

1 Does not allow any customisation or requires setting to be input every time

2 Allows little customisation and that limits app’s functions

3 Basic customisation to function adequately

4 Allows numerous options for customisation

5 Allows complete tailoring the user’s characteristics/preferences, remembers all settings

1. **Does it allow user input, provide feedback, contain prompts (reminders, sharing options, notifications, etc.)?**

1 No interactive features and/or no response to user input

2 Some, but not enough interactive features which limits app’s functions

3 Basic interactive features to function adequately

4 Offers a variety of interactive features, feedback and user input options

5 Very high level of responsiveness through interactive features, feedback and user input options

1. **Is the app content (visuals, language, design) appropriate for the target audience?**

1 Completely inappropriate, unclear or confusing

2 Mostly inappropriate, unclear or confusing

3 Acceptable but not specifically designed for the target audience. May be inappropriate/unclear/confusing at times

4 Designed for the target audience, with minor issues

5 Designed specifically for the target audience, no issues found

1. **How accurately/fast do the app features (functions) and components (buttons/menus) work?**

1 App is broken; no/insufficient/inaccurate response (e.g. crashes/bugs/broken features, etc.)

2 Some functions work, but lagging or contains major technical problems

3 App works overall. Some technical problems need fixing, or is slow at times

4 Mostly functional with minor/negligible problems

5 Perfect/timely response; no technical bugs found, or contains a ‘loading time left’ indicator (if relevant)

1. **How easy is it to learn how to use the app; how clear are the menu labels, icons and instructions?**

1 No/limited instructions; menu labels, icons are confusing; complicated

2 Takes a lot of time or effort

3 Takes some time or effort

4 Easy to learn (or has clear instructions)

5 Able to use app immediately; intuitive; simple (no instructions needed)

1. **Does moving between screens make sense; Does app have all necessary links between screens?**

1 No logical connection between screens at all /navigation is difficult

2 Understandable after a lot of time/effort

3 Understandable after some time/effort

4 Easy to understand/navigate

5 Perfectly logical, easy, clear and intuitive screen flow throughout, and/or has shortcuts

1. **Do taps/swipes/pinches/scrolls make sense? Are they consistent across all components/screens?**

1 Completely inconsistent/confusing

2 Often inconsistent/confusing

3 OK with some inconsistencies/confusing elements

4 Mostly consistent/intuitive with negligible problems

5 Perfectly consistent and intuitive

1. **Is arrangement and size of buttons, icons, menus and content on the screen appropriate?**

1 Very bad design, cluttered, some options impossible to select, locate, see or read

2 Bad design, random, unclear, some options difficult to select/locate/see/read

3 Satisfactory, few problems with selecting/locating/seeing/reading items

4 Mostly clear, able to select/locate/see/read items

5 Professional, simple, clear, orderly, logically organised

1. **How high is the quality/resolution of graphics used for buttons, icons, menus and content?**

1 Graphics appear amateur, very poor visual design - disproportionate, stylistically inconsistent

2 Low quality/low resolution graphics; low quality visual design – disproportionate

3 Moderate quality graphics and visual design (generally consistent in style)

4 High quality/resolution graphics and visual design – mostly proportionate, consistent in style

5 Very high quality/resolution graphics and visual design - proportionate, consistent in style throughout

1. **How good does the app look?**

1 Ugly, unpleasant to look at, poorly designed, clashing, mismatched colours

2 Bad – poorly designed, bad use of colour, visually boring

3 OK – average, neither pleasant, nor unpleasant

4 Pleasant – seamless graphics – consistent and professionally designed

5 Beautiful – very attractive, memorable, stands out; use of colour enhances app features/menus

1. **Is app content correct, well written, and relevant to the goal/topic of the app?**

1 Irrelevant/inappropriate/incoherent/incorrect

2 Poor. Barely relevant/appropriate/coherent/may be incorrect

3 Moderately relevant/appropriate/coherent/and appears correct

4 Relevant/appropriate/coherent/correct

5 Highly relevant, appropriate, coherent, and correct

1. **Is the information within the app comprehensive but concise?**

1 Minimal or overwhelming

2 Insufficient or possibly overwhelming

3 OK but not comprehensive or concise

4 Offers a broad range of information, has some gaps or unnecessary detail; or has no links to more information and resources

5 Comprehensive and concise; contains links to more information and resources

1. **Is visual explanation of concepts – through charts/graphs/images/videos, etc. – clear, logical, correct?**

1 Completely unclear/confusing/wrong or necessary but missing

2 Mostly unclear/confusing/wrong

3 OK but often unclear/confusing/wrong

4 Mostly clear/logical/correct with negligible issues

5 Perfectly clear/logical/correct

1. **Does the information within the app seem to come from a credible source?**

1 Suspicious source

2 Lacks credibility

3 Not suspicious but legitimacy of source is unclear

4 Possibly comes from a legitimate source

5 Definitely comes from a legitimate/specialised source

1. **Would you recommend this app to people who might benefit from it?**

1 Not at all I would not recommend this app to anyone

2 There are very few people I would recommend this app to

3 Maybe There are several people I would recommend this app to

4 There are many people I would recommend this app to

5 Definitely I would recommend this app to everyone

1. **How many times do you think you would use this app in the next 12 months if it was relevant to you?**

| 1  None | 2  1-2 | 3  3-10 | 4  10-50 | 5  >50 |
| --- | --- | --- | --- | --- |

1. **Would you pay for this app?**

| 1  Definitely not | 2 | 3 | 4 | 5  Definitely yes |
| --- | --- | --- | --- | --- |

1. **The app provides an acceptable way to deliver health care services.**

| 1  Not at all | 2 | 3 | 4 | 5  Highly |
| --- | --- | --- | --- | --- |

1. **The app will be useful for health care practice.**

| 1  Not at all | 2 | 3 | 4 | 5  Highly |
| --- | --- | --- | --- | --- |

1. **The app improves access to delivering health care services.**

| 1  Not at all | 2 | 3 | 4 | 5  Highly |
| --- | --- | --- | --- | --- |

1. **The app helps to manage patients’ health effectively.**

| 1  Not at all | 2 | 3 | 4 | 5  Highly |
| --- | --- | --- | --- | --- |

1. **The app provides an acceptable way to deliver health care services, such as accessing educational materials, tracking patients’ activities and self-assessment.**

| 1  Not at all | 2 | 3 | 4 | 5  Highly |
| --- | --- | --- | --- | --- |

1. **What is your overall (star) rating of the app?**

| 1 *  One of the worst apps I’ve used | 2 ** | 3 ***  Average | 4 **** | 5 *****  One of the best apps I’ve used |
| --- | --- | --- | --- | --- |

**Section D: Please rate the overall content of the app based on the 4-point scale below. Please tick (√) in the appropriate box based on your rating for the four criteria.**

|  | **Criteria** | **Score** | **Overall rating** |
| --- | --- | --- | --- |
| 1 | Relevance | 1= not relevant |  |
|  |  | 2= items need some revision |  |
|  |  | 3= relevant but need minor revision |  |
|  |  | 4= very relevant |  |
| 2 | Clarity | 1= not clear |  |
|  |  | 2= items need some revision |  |
|  |  | 3= clear but need minor revision |  |
|  |  | 4= very clear |  |
| 3 | Simplicity | 1= not simple |  |
|  |  | 2= items need some revision |  |
|  |  | 3= simple but need minor revision |  |
|  |  | 4= very simple |  |
| 4 | Ambiguity | 1= doubtful |  |
|  |  | 2= items need some revision |  |
|  |  | 3= no doubt but need minor revision |  |
|  |  | 4= meaning is clear |  |

| **Further comments about the app?** |
| --- |

# Supplementary Material C. *FamNucleus* User Evaluations: Focus Group Discussion (FGD) Guide

*This guide is not a script and is intended to be used flexibly, with participant responses guiding the flow of the conversation, topics covered in the order that they naturally arise, and probes used as appropriate. Not all of the questions in this discussion guide may be asked during each FGD.* *However, moderators will consistently reference objectives at the top of each section, so all key information requirements will be met.*

| Black: Main Discussion Guide \| Red: For moderator to take note | | |
| --- | --- | --- |
| **Section A: Warm-up & context setting (10 mins)**  To build rapport and ease participants into the discussion. | | |
| A.1 | **Moderator introduction** | 1. Thank respondents for their time. Check video and sound. 2. Introduction, study background / purpose: Understand your experience of using the *FamNucleus* app prototype, and gather your ideas on how to make it more useful for you and your family. 3. No right / wrong answers, value honest views. 4. Speaking one at a time. Equal participation encouraged. 5. Emphasise confidentiality: No recordings will be shared publicly; all findings will be aggregated and anonymised. 6. 1.5hr discussion length. |
| A.2 | **Participant introduction** | 1. Self-introduction: Name, occupation, family composition, infant’s age, parenting experience (1^st^-time / experienced). 2. Ice-breaker question: If could describe your parenting journey in 3 words, how would you describe it? |

| **Section B: Expectations and initial impressions (20 mins)**  To explore parents' motivations, expectations and early experiences with the app, and understand the competitive landscape. | | |
| --- | --- | --- |
| B.1 | **Expectations** | **Before today’s group discussion, you downloaded and tested out a prototype for a new parenting app called *FamNucleus*.**   1. Before using *FamNucleus*, how did you manage your baby’s needs and track their growth? What other apps, tools or resources did you rely on? 2. What drew you to try *FamNucleus*? What did you hope it would help you with? |
| B.2 | **First impressions** | **Here are some screenshots of the app for you to refer to, as we to dive into your experiences and feedback.**  *Moderator to flash slide with* ***key app features***.   1. Thinking back to when you first downloaded *FamNucleus*…    1. What stood out most – positive or negative?    2. Which features looked most promising?    3. Any features that seemed unnecessary or confusing? 2. Can you walk us through your first interactions with the app?    1. How easy / difficult did you find the initial setup?    2. What was the first thing you did after setting up your profile?    3. Any immediate surprises (positive / negative)?    4. What kept you engaged (or what made you stop)? 3. Did FamNucleus meet, exceed or fall short of what you expected? In what ways? (Probe for specific examples.) 4. How motivated were you to keep exploring and using the app? |

| **Section C: Feature and nudge deep-dive (40 mins)**  To evaluate the usefulness, usability and improvement areas of app features and nudges. | | |
| --- | --- | --- |
| C.1 | **Feedback on app features** | *Moderator to provide each participant with a set of* ***feature cards*** *(*Quest; Diary / chatbot; Enrich – Videos, articles, infographics, events, helplines, nudges; Forum; Survey / tracker) and ***2x2 matrix template*** *(*Most to least valuable; Easiest to most difficult to use).  *Participants to sort features on the matrix, mark their most used features with stars, and set aside any features they did not try out.*   1. Which features did you use the most during the 4wk trial? Why? 2. Were there any features you didn’t use? If yes, why? 3. Why did you place features where you did on the 2x2 matrix? 4. [For each feature] What would make this feature…    1. More useful to you?    2. Easier for you to use? 5. What features are missing that would be valuable to you? |
| C.2 | **Feedback on nudges** | *Moderator to show actual* ***nudge messages*** *sent to participants****.***   1. Do you remember receiving notifications or reminders from the app? Which ones stood out? 2. [For each nudge message]    1. Relevance – How relevant was this to your situation?    2. Clarity – How easy was it to understand? Any unclear words / phrases?    3. Impact - Did it lead you to take any action or do anything differently? Why or why not? 3. What types of nudges would you find most useful? 4. When and how often would you prefer to receive them? 5. Via what format (e.g. in-app vs push notification)? 6. Any other suggestions to make nudges more relevant and effective for you, e.g. length, tone, design? |

| **Section D: App engagement (15 mins)**  To understand user engagement patterns and ways to drive sustained usage. | | |
| --- | --- | --- |
| D.1 | **Usage patterns & daily integration** | *Moderator to provide each participant with a timeline template and ask them to map out their typical day, and when they interacted with FamNucleus.*   1. What triggered you to open the app? 2. What prevented you from using it more? 3. How well does the app fit into your daily routine? 4. How did the app complement, replace or compete with other parenting tools / resources you used? 5. How did / might you use it during healthcare visits or interactions with healthcare professionals? |
| D.2 | **Future use** | 1. What would make you want to use this app more? 2. How likely are you to continue using the app? Why? 3. What would make you recommend it to other parents? What would it’s unique selling point (USP) be? |

| **Section E: Conclusion (5 mins)**  To capture final thoughts or questions. | | |
| --- | --- | --- |
| E.1 | **Final thoughts and wrap up** | *[Moderator to go round each participant]*   1. What’s the 1 thing you would add /change to make *FamNucleus* your go-to parenting tool? 2. Any other thoughts or feedback we haven’t covered? 3. Thank you for your time and valuable feedback, which will be used to enhance the app before it launches. |

# Supplementary Material D: Detailed evaluations of the UEQ for MVP phase

*
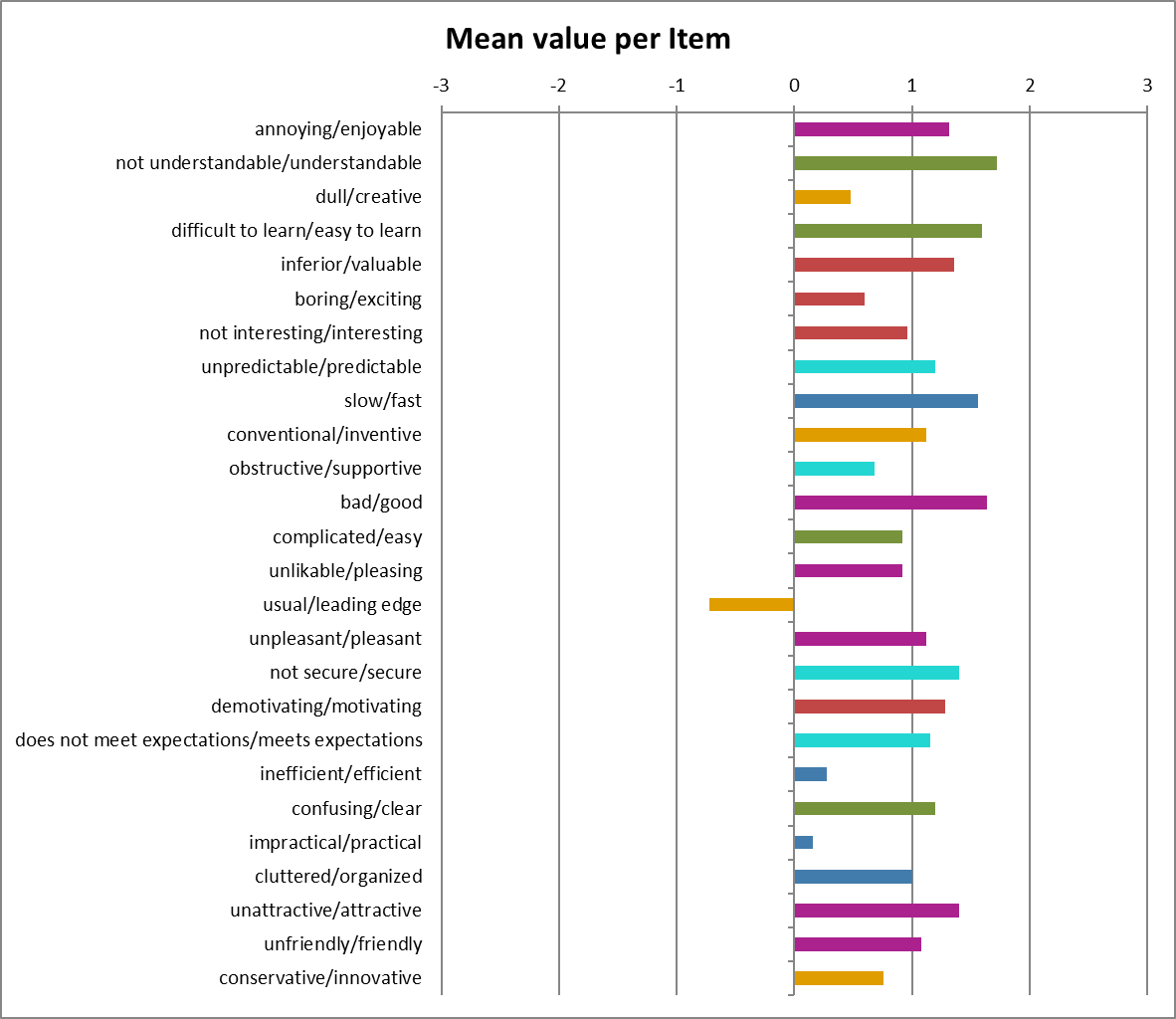
***Supplementary Figure 1. Mean values per item of the User Experience Questionnaire (UEQ) for parents.**

**Supplementary Figure 1.** Each bar represents the average rating for an individual item on a scale from –3 (most negative evaluation) to +3 (most positive evaluation). Positive values reflect more favorable user experiences, while negative values indicate less favorable evaluations.

**Table 2. Overall results of the UEQ [-3,3] from parents (n=25).**

| **Scale** | **Mean** | **Standard Deviation** | **Confidence** | **Confidence interval** | |
| --- | --- | --- | --- | --- | --- |
| **Attractiveness** | 1.247 | 0.967 | 0.379 | 0.868 | 1.626 |
| **Perspicuity** | 1.360 | 0.916 | 0.359 | 1.001 | 1.719 |
| **Efficiency** | 0.750 | 0.974 | 0.382 | 0.368 | 1.132 |
| **Dependability** | 1.110 | 1.111 | 0.436 | 0.674 | 1.546 |
| **Stimulation** | 1.050 | 1.161 | 0.455 | 0.595 | 1.505 |
| **Novelty** | 0.410 | 1.007 | 0.395 | 0.015 | 0.805 |

*
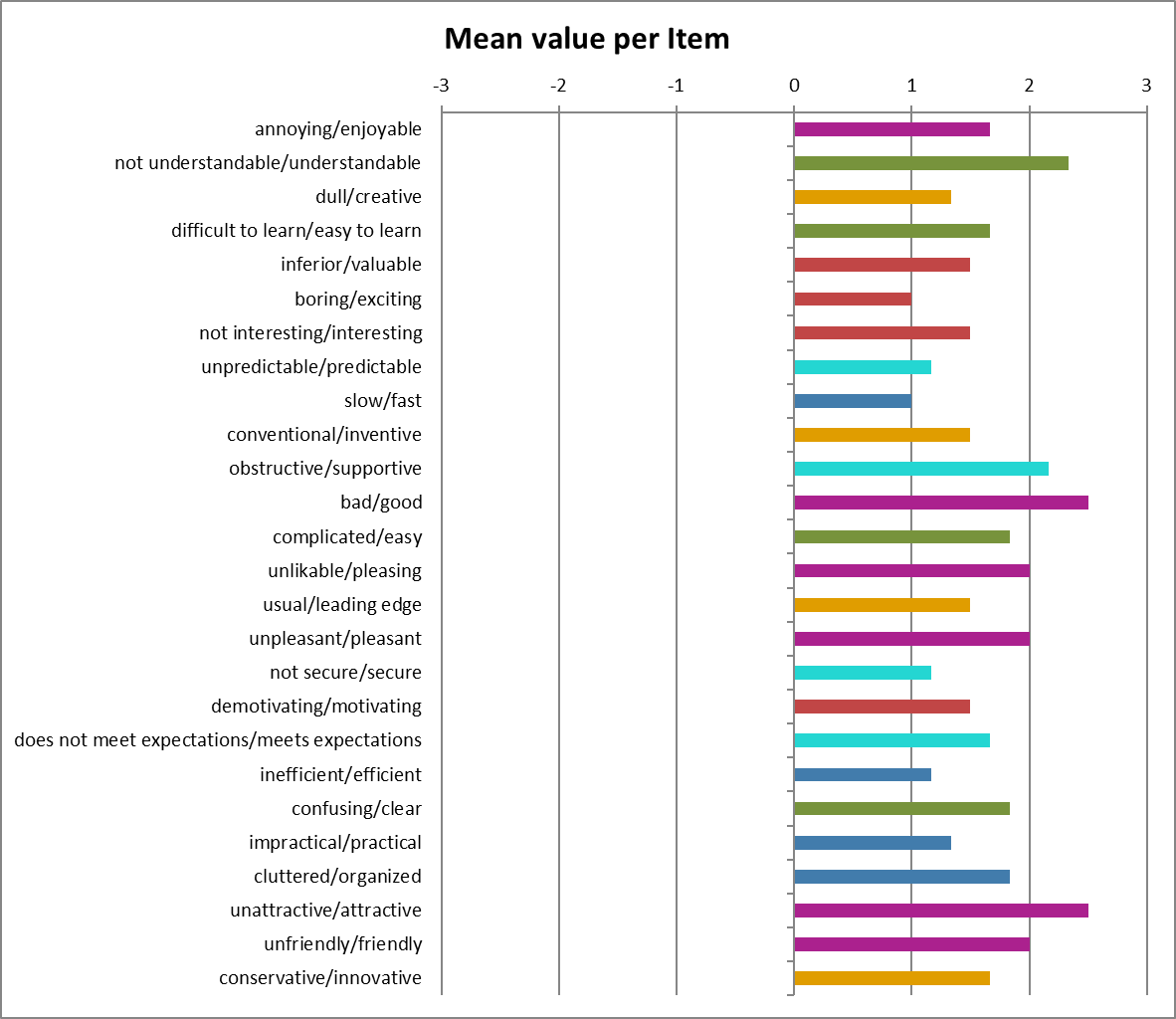
***Supplementary Figure 2. Mean values per item of the User Experience Questionnaire (UEQ) for parents.**

**Supplementary Figure 2.** Each bar represents the average rating for an individual item on a scale from –3 (most negative evaluation) to +3 (most positive evaluation). Positive values reflect more favorable user experiences, while negative values indicate less favorable evaluations.

| **Scale** | **Mean** | **Standard Deviation** | **Confidence** | **Confidence interval** | |
| --- | --- | --- | --- | --- | --- |
| **Attractiveness** | 2.111 | 0.750 | 0.600 | 1.511 | 2.711 |
| **Perspicuity** | 1.917 | 0.944 | 0.756 | 1.161 | 2.672 |
| **Efficiency** | 1.333 | 1.310 | 1.048 | 0.285 | 2.382 |
| **Dependability** | 1.542 | 0.697 | 0.557 | 0.984 | 2.099 |
| **Stimulation** | 1.375 | 0.542 | 0.434 | 0.941 | 1.809 |
| **Novelty** | 1.500 | 1.140 | 0.912 | 0.588 | 2.412 |

**Table 2. Overall results of the UEQ [-3,3] from HCPs (n=6).**

**Supplementary Material E: Mean, standard deviation (SD), minimum and maximum values for the scales and subscales of the Mobile Application Rating Scale: User Version (uMARS)**

|  | **Parents** | | | | **HCPs** | | | |
| --- | --- | --- | --- | --- | --- | --- | --- | --- |
| **Subscales** | **Mean** | **SD** | **Min.** | **Max.** | **Mean** | **SD** | **Min.** | **Max.** |
| **Section A-Engagement** | **3.59** | **0.68** | **2.20** | **4.60** | **3.63** | **0.56** | **3.00** | **4.4** |
| Entertainment | 3.64 | 0.86 | 2.00 | 5.00 | 4.00 | 0.89 | 3.00 | 5.00 |
| Interest | 3.52 | 0.83 | 2.00 | 5.00 | 3.83 | 0.75 | 3.00 | 5.00 |
| Customisation | 3.48 | 1.36 | 1.00 | 5.00 | 3.00 | 1.41 | 1.00 | 5.00 |
| Interactivity | 3.44 | 1.08 | 1.00 | 5.00 | 3.33 | 0.82 | 2.00 | 4.00 |
| Target group | 3.88 | 0.73 | 2.00 | 5.00 | 4.00 | <0.001 | 4.00 | 4.00 |
| **Section B-Functionality** | **4.07** | **0.44** | **3.25** | **4.5** | **4.04** | **0.40** | **3.50** | **4.50** |
| Performance | 3.76 | 0.72 | 2.00 | 5.00 | 3.67 | 0.52 | 3.00 | 4.00 |
| Ease of use | 4.12 | 0.67 | 3.00 | 5.00 | 4.17 | 0.75 | 3.00 | 5.00 |
| Navigation | 4.32 | 0.80 | 3.00 | 5.00 | 4.17 | 0.75 | 3.00 | 5.00 |
| Gestural design | 4.08 | 0.64 | 3.00 | 5.00 | 4.17 | 0.41 | 4.00 | 5.00 |
| **Section C- Aesthetics** | **4.12** | **0.60** | **2.67** | 5.00 | **4.39** | **0.49** | **3.67** | **5.00** |
| Layout | 4.24 | 0.83 | 3.00 | 5.00 | 4.33 | 0.82 | 3.50 | 4.50 |
| Graphics | 3.96 | 0.89 | 2.00 | 5.00 | 4.33 | 0.52 | 3.00 | 5.00 |
| Visual appeal | 4.16 | 0.75 | 3.00 | 5.00 | 4.50 | 0.55 | 4.00 | 5.00 |
| **Section D-Information** | **4.19** | **0.42** | **3.00** | **4.75** | **4.21** | **0.37** | **3.75** | **4.75** |
| Quality of information - Content relevance | 4.12 | 0.73 | 3.00 | 5.00 | 4.17 | 0.41 | 4.00 | 5.00 |
| Quantity of information - Comprehensive but concise | 3.84 | 0.69 | 2.00 | 5.00 | 3.83 | 0.75 | 3.00 | 5.00 |
| Visual information | 4.48 | 0.59 | 3.00 | 5.00 | 4.33 | 0.52 | 4.00 | 5.00 |
| Credibility of source | 4.32 | 0.75 | 3.00 | 5.00 | 4.50 | 0.54 | 4.00 | 5.00 |
| **Section E-Subjective quality** | **3.18** | **0.54** | **2.00** | **4.00** | **3.58** | **0.63** | **2.75** | **4.50** |
| Recommendation to others | 3.92 | 0.91 | 2.00 | 5.00 | 4.17 | 0.75 | 3.00 | 5.00 |
| Frequency of app usage in 12 months | 3.48 | 0.87 | 2.00 | 5.00 | 3.67 | 1.03 | 2.00 | 5.00 |
| Willingness to pay for the app | 1.96 | 1.18 | 1.00 | 4.00 | 2.50 | 1.05 | 1.00 | 4.00 |
| Overall rating of the app | 3.36 | 0.76 | 2.00 | 4.00 | 4.00 | 0.63 | 3.00 | 5.00 |
| **Section F-Perceived impact** | 3.60 | 0.59 | 2.33 | 4.83 |  |  |  |  |
| This app has increased my awareness of the importance of addressing the health behaviour. | 3.52 | 0.59 | 3.00 | 5.00 |  |  |  |  |
| This app has increased my knowledge/understanding of the health behaviour. | 3.64 | 0.64 | 3.00 | 5.00 |  |  |  |  |
| The app has changed my attitudes toward improving the health behaviour. | 3.60 | 0.76 | 2.00 | 5.00 |  |  |  |  |
| The app has increased my intentions/motivation to address the health behaviour. | 3.64 | 0.64 | 3.00 | 5.00 |  |  |  |  |
| This app would encourage me to seek further help to address the health behaviour (if I needed it). | 3.72 | 0.89 | 1.00 | 5.00 |  |  |  |  |
| Use of this app will increase/decrease the health behaviour in my family. | 3.48 | 1.05 | 1.00 | 5.00 |  |  |  |  |

**Descriptive data on subjective quality rated by parents and HCPs:**

| **Subjective quality evaluation** | **Frequency (%)** | |
| --- | --- | --- |
|  | **Parents (n=25)** | **HCPs (n=6)** |
| Recommendation to others ^a^  Low  Neutral  High | 1 (4.0)  8 (32.0)  16 (64.0) | 0 (0)  1 (16.7)  5 (83.3) |
| Frequency of app usage in 12 months ^b^  Low  Moderate  High  Very high | 3 (12.0)  10 (40.0)  9 (36.0)  3 (12.0) | 1 (16.7)  11 (16.7)  3 (50.0)  1 (16.7) |
| Willingness to pay for the app ^a^  Low  Neutral  High | 19 (76.0)  1 (4.0)  5 (20.0) | 3 (50.0)  2 (33.3)  1 (16.7) |
| Overall rating of the app ^a^  Low  Neutral  High | 4 (16.0)  8 (32.0)  13 (52.0) | 0 (0)  1 (16.7)  5 (83.3) |

^a^ Recommendation intention, willingness to pay for the app, and overall rating of the app were recoded into three categories: Low (scores 1-2), Neutral (score 3), and High (scores 4-5).

^b^ Frequency of app usage were recoded into three categories: Low (1-2 times), Moderate (3-5 times), High (10-50 times), and Very high (>50 times).
